# The Novel Nucleoside Analogue ProTide NUC-7738 Overcomes Cancer Resistance Mechanisms *In Vitro* and in a First-In-Human Phase I Clinical Trial

**DOI:** 10.1158/1078-0432.CCR-21-1652

**Published:** 2021-12-01

**Authors:** Hagen Schwenzer, Erica De Zan, Mustafa Elshani, Ruud van Stiphout, Mary Kudsy, Josephine Morris, Valentina Ferrari, In Hwa Um, James Chettle, Farasat Kazmi, Leticia Campo, Alistair Easton, Sebastian Nijman, Michaela Serpi, Stefan Symeonides, Ruth Plummer, David J. Harrison, Gareth Bond, Sarah P. Blagden

**Affiliations:** 1 Department of Oncology, Medical Sciences Division, University of Oxford, Oxford, United Kingdom.; 2 Ludwig Institute for Cancer Research, Nuffield Department of Medicine, University of Oxford, Oxford, United Kingdom.; 3 Target Discovery Institute, Nuffield Department of Medicine, University of Oxford, Oxford, United Kingdom.; 4 School of Medicine, University of St Andrews, St. Andrews, United Kingdom.; 5 School of Pharmacy and Pharmaceutical Sciences, University of Cardiff, Cardiff, United Kingdom.; 6 Cancer Research UK Edinburgh Centre, Institute of Genetics and Cancer, The University of Edinburgh, Western General Hospital, Edinburgh, United Kingdom.; 7 Northern Centre for Cancer Care, Newcastle Hospitals NHS Foundation Trust, Freeman Hospital, Newcastle upon Tyne, United Kingdom.; 8 NuCana PLC, Edinburgh, United Kingdom.

## Abstract

**Purpose:**

Nucleoside analogues form the backbone of many therapeutic regimens in oncology and require the presence of intracellular enzymes for their activation. A ProTide is comprised of a nucleoside fused to a protective phosphoramidate cap. ProTides are easily incorporated into cells whereupon the cap is cleaved and a preactivated nucleoside released. 3′-Deoxyadenosine (3′-dA) is a naturally occurring adenosine analogue with established anticancer activity *in vitro* but limited bioavailability due to its rapid *in vivo* deamination by the circulating enzyme adenosine deaminase, poor uptake into cells, and reliance on adenosine kinase for its activation. In order to overcome these limitations, 3′-dA was chemically modified to create the novel ProTide NUC-7738.

**Experimental Design:**

We describe the synthesis of NUC-7738. We determine the IC_50_ of NUC-7738 using pharmacokinetics (PK) and conduct genome-wide analyses to identify its mechanism of action using different cancer model systems. We validate these findings in patients with cancer.

**Results:**

We show that NUC-7738 overcomes the cancer resistance mechanisms that limit the activity of 3′-dA and that its activation is dependent on ProTide cleavage by the enzyme histidine triad nucleotide-binding protein 1. PK and tumor samples obtained from the ongoing first-in-human phase I clinical trial of NUC-7738 further validate our *in vitro* findings and show NUC-7738 is an effective proapoptotic agent in cancer cells with effects on the NF-κB pathway.

**Conclusions:**

Our study provides proof that NUC-7738 overcomes cellular resistance mechanisms and supports its further clinical evaluation as a novel cancer treatment within the growing pantheon of anticancer ProTides.

**Translational Relevance**

ProTide modification of nucleoside analogues is designed to overcome the cellular resistance mechanisms that limit their efficacy. This consists of the chemical addition of a protective phosphoramidate moiety onto the parent nucleoside, in this case the natural nucleoside analogue 3′-deoxyadenosine (3′-dA) to form the ProTide NUC-7738. The mode of action of 3′-dA has been the subject of speculation as its major limitation is its short plasma half-life due to rapid enzymatic deamination by adenosine deaminase (ADA). We demonstrate that NUC-7738 is resistant to deamination by ADA and is cleaved by the intracellular phosphoramidase HINT1 into 3′-dAMP for conversion to the active metabolites 3′-dADP and 3′-dATP. We show that NUC-7738 promotes proapoptotic pathways and attenuates NF-κB. These findings are validated in tumor samples from patients in an ongoing first-in-human trial of NUC-7738 from which we have identified biomarkers to enrich our understanding of NUC-7738 and patients most likely benefiting from it.

## Introduction

3′-deoxyadenosine (3′-dA) or cordycepin is a natural nucleoside analogue and the bioactive component of the fungus *Cordyceps sinensis*, a traditional remedy for inflammatory diseases and cancer (1–6). The pharmacologic activity of 3′-dA is attributed to its major metabolite, 3′-dA-5′-triphosphate [3′-dATP or cordycepin triphosphate (CTP); refs. 1, 2, 7], which is generated when 3′-dA is converted to 3′-deoxyadenosine monophosphate (3′-dAMP) by the rate-limiting phosphotransferase adenosine kinase (ADK), and from thence to 3′-deoxyadenosine diphosphate (3′-dADP) and 3′-dATP. The drug has proven pleotropic *in vitro* activities including mRNA transcription inhibition (8) and depletion via poly(A)-tail length destabilisation (9, 10), activation of AMPK (11), and induction of apoptosis (6, 12–15). Despite these potent *in vitro* effects, it has minimal *in vivo* activity due to its rapid hydrolysis by the ubiquitous circulating enzyme adenosine deaminase (ADA) and poor uptake into cells via the nucleoside transporter hENT1 (11, 16). To improve its stability 3′-dA has been coadministered with the ADA inhibitor pentostatin in preclinical studies and two clinical trials (NCT0003005 and NCT00709215) but the combination induces synergistic and dose-limiting toxicities such as hepatic and renal damage (16–19). Like a selection of other nucleoside analogues, the structure and stepwise phosphorylation of 3′-dA makes it a potential candidate for ProTide chemistry. Using this approach, a preactivated or monophosphate version is chemically synthesized and fused to a protective cap, comprised of an aryl, ester, and amino acid group termed the phosphoramidate motif to form the ProTide. Once inside the cell, the motif is hydrolyzed releasing the active drug. ProTide chemistry has been applied to nucleoside antiviral therapies to generate sofosbuvir and to the anti-cancer nucleoside analogues 5′-fluorouracil (5′-FU; refs. 20, 21) and gemcitabine to generate NUC-3373 and NUC-1031 (Acelarin), respectively. ProTides are clinically attractive as they bypass the rate-limiting steps of nucleoside uptake and monophosphorylation in order to deliver a high concentration of active drug into cells (22–27). In the case of 3′-dA, ProTide modification is anticipated to provide the additional benefit of protection from ADA degradation. Here we describe the phosphoramidate transformation of 3′-dA to the ProTide NUC-7738, and the RNA sequencing (RNA-seq) and genome-wide genetic screens conducted to assess the mechanism of activation and activity of NUC-7738 compared with the parent drug, 3′-dA. A first-in-human (phase I) dose-escalation and -expansion trial of NUC-7738 commenced in June 2019 and is currently ongoing. Pharmacokinetic (PK) and tumor samples obtained from study participants further validate our *in vitro* findings that NUC-7738 is an effective proapoptotic agent in cancer cells with additional effects on the NF-κB pathway.

## Materials and Methods

### Drug synthesis

Briefly, to prepare compound **12**, 3′dA was protected quantitatively at the 2′ and 3′ position with *tert*-butyl dimethylsilyl chloride in N,N-dimethylmethanamide (DMF) in the presence of imidazole and 4-dimethylaminopyridine (DMAP) to obtain compound **10**. Selective 5′-deprotection of the silyl group with a 1:1:4 mixture of trifluoroacetic acid (TFA) in water and tetrahydrofuran (THF) yielded 98% of desired product **11**. Coupling of compound **11** with phenyl L-alanine benzyl ester phosphorochloridate by means of 1-methylimidazole (NMI) in THF yielded 62% of 2′-TBDMS (tert-butyldimethylsilyl ether) protected ProTide **12** (28). Deprotection of **12** using TFA in dichloromethane yielded ProTide **7** in good yield. This protection–deprotection strategy proved to be an efficient way to prepare **7a** obtained with an overall yield of 42%. Chemical synthesis of 3′-deoxyadenosine-5′-O-phenyl-(benzyloxy-L-alaninyl)-phosphate and its precursors: 2′,5′-(bis-O-tert-butyldimethylsilyl)3′-deoxyadenosine (10), 2′-O-tert-butyldimethylsilyl-3′-deoxyadenosine (11), and 3′-deoxyadenosine-2′-O-[tert-butyldimethylsilyl-5′-phenyl-(benzyloxy-L-alaninyl)]-phosphate (12) was performed as detailed below.

#### 2′,5′-(bis-O-tert-butyldimethylsilyl)3′-deoxyadenosine (compound 10)

3′-Deoxyadenosine (ref. 1; 1.13 g, 4.5 mmol/L), was dissolved in DMF (50 mL) and tert-butyldimethylsilyl chloride (TBDMSCI; 2.03 g, 13.5 mmol/L) and imidazole (1.84 g, 27 mmol/L) were added to the flask. The mixture was stirred for 16 hours at room temperature. The reaction mixture was diluted with CHCl_3_ (20 mL) and the organics were washed with NH_4_Cl (saturated solution, 20 mL x 3). The organics were dried over Na_2_SO_4_, filtered, and evaporated to afford **10** as a sticky solid (1.56 g, 72%).

#### 2′-O-tert-butyldimethylsilyl-3′-deoxyadenosine (compound 11)

Compound **10** (1.56 g, 3.25 mmol/L) was dissolved in THF (2 mL) and cooled down to 0°C. A solution of TFA in water (1 mL, 1/1 v/v) was added dropwise and the mixture was stirred for 5 hours at 0°C. The solution was evaporated under vacuum and the crude purified via Biotage Isolera One [30 g ZIP cartridge KP SIL, 60 mL/minute, gradient eluent system 2%–20% CH_3_OH/CH_2_Cl_2_ 10CV (column volume), 20% 5CV] to yield **11** as a white foam (1.16 g, 98%).

#### 3′-deoxyadenosine-2′-O-[tert-butyldimethylsilyl-5′-phenyl-(benzyloxy-L-alaninyl)]-phosphate (compound 12)

To a solution of 2′-*O*-*tert*-butyldimethylsilyl-3′-deoxyadenosine **11** (0.05 g, 0.14 mmol/L) in THF (2 mL), phenyl-(benzyloxy-L- alaninyl) phosphorochloridate (ref. 28; 0.14 g, 0.41 mmol/L) in THF (0.5 mL) and *N*-methylimidazole (56 μL, 0.7 mmol/L) were added. The crude was purified via Biotage Isolera One (10 g SNAP cartridge KP SIL, 10 mL/minute, gradient eluent system 1%–10% CH_3_OH/CH_2_Cl_2_ 12CV, 10% 2CV) to afford the title compound **12** as a white solid (0.06 g, 62%).

#### 3′-deoxyadenosine 5′-O-phenyl-(benzyloxy-L-alaninyl)-phosphate (compound 7):

Compound **12** (0.10 g, 0.14 mmol/L) was dissolved in anhydrous CH_2_Cl_2_ (2 mL), and cooled down at 0°C. TFA (2 mL) was added dropwise to this solution and the reaction stirred overnight at room temperature. Purification by Biotage Isolera One [cartridge SNAP 25 g, 25 mL/minute, CH_3_OH/CH_2_Cl_2_ 1%–8% 10 CV, 8% 5 CV) afforded the title compound as a white solid (0.063 g, 76%). Reverse-phase high-performance liquid chromatography (HPLC) eluting with H_2_O/CH_3_CN from 100/10 to 0/100 in 30 minutes, F = 1 mL/minute, λ = 254 nm, t_R_ 13.56 and 13.75 minutes. C_26_H_29_N_6_O_7_P required *m/z* 568.2 [M]. Electrospray (ES) ionisation mass spectrometry (in ES+ mode) found *m/z* 569.2 [M+H]^+^, 591.2 [M+Na]^+^, 1,159.4 [2M+Na]^+^.

All solvents used were anhydrous and used as supplied by Sigma-Aldrich. All commercially available reagents were supplied by either Sigma-Aldrich or Fisher and used without further purification. All solid reagents were dried for several hours under high vacuum prior to use. For analytical thin-layer chromatography (TLC), precoated aluminium-backed plates (60 F-54, 0.2-mm thickness; supplied by E. Merck AG) were used and developed by an ascending elution method. After solvent evaporation, compounds were detected by quenching of the fluorescence, at 254 nm upon irradiation with a UV lamp. Column chromatography purifications were carried out by means of automatic Biotage Isolera One. Fractions containing the product were identified by TLC and pooled, and the solvent was removed in vacuo. ^1^H, ^31^P, and ^13^C nuclear magnetic resonance (NMR) spectra were recorded in a Bruker Avance 500 spectrometer at 500 MHz, 202 MHz, and 125 MHz, respectively and autocalibrated to the deuterated solvent reference peak in case of ^1^H and ^13^C NMR and 85% H_3_PO_4_ for ^31^P experiments (see Supplementary Materials). All ^31^P and ^13^C NMR spectra were proton-decoupled. Analytical HPLC analysis was performed using both Spectra System SCM (with X-select-C18, 5 mm, 4.8×150–mm column) and Varian Prostar system (LCWorkstation- Varian Prostar 335 LC detector). Low-resolution mass spectrometry was performed on a Bruker Daltonics micrOTOF^TM^-LC system (atmospheric pressure ionization, electron spray mass spectroscopy) in positive mode. Of note, all compounds used for the preclinical investigation were synthesized at a small scale yielding in 50 to 100 mg product per batch. The purity more than 99% of the final compound **7** was confirmed using HPLC analysis and prepared at a facility under Good Manufacturing Product (GMP) guidelines for use in clinical research studies.

### Cell culture

HAP1 cells were grown in Iscove's Modified Dulbecco's Medium. Stomach adenocarcinoma cell line (AGS) cells were grown in Ham's F-12 Medium. CAKI-1 (RRID:CVCL_0234), NCI-786 (RRID:CVCL_1051 A498), UO-31 (RRID:CVCL_1911), ACHN (RRID:CVCL_1067), 501MEL (RRID:CVCL_4633), OVCAR-8 (RRID:CVCL_1629), SK-OV3 (RRID:CVCL_0532), and Tera-1 cells (RRID:CVCL_2776) were cultivated in RPMI 1640 Medium. HeLa cells (RRID:CVCL_0030) were grown in DMEM with high glucose. All media were supplemented with 10% FCS and 1% penicillin G and streptomycin. Cells were cultivated at 37°C and 5% CO_2_ in a humidified cell culture incubator for no longer than 15 passages after thawing.

### Cellular function assays

Cells (10^4^) were seeded into 96-well plates and then treated with NUC-7738 (lot 5132/E00393/119) or 3′-dA (cordycepin, catalog no. C3394, lot 067M4182, Sigma) for 48 hours. One and a half milligrams per milliliter MTT (catalog no. M6494, Invitrogen) in a 1:1 ratio was added to cell culture media. After a 2-hour incubation at 37°C, media was removed and 70 μl of isopropanol was added. Optical densities at 570 nm were measured using POLARstar Omega plate reader. Readouts were normalized and IC_50_ values were calculated using a nonlinear regression model in Graphpad prism (v8.3.0). Each biological replicate (at least 3) was performed in 4 technical replicates to calculate the average IC_50_ value. To measure apoptosis, the Guava NexinReagent (Luminex Corp.) was used following manufacturer's instructions. Fluorescence staining was measured with an automated 96-well Guava easyCyte Flow Cytometer (Luminex Corp.).

### Haploid genetic screen

Gene-trap mutagenesis of wild-type (WT) HAP1 cells was performed as described previously (29–31). An initial mutant library (31) containing 20 million HAP1 cells were seeded in a T175 flask and expanded to 250 million cells. For each treatment group, cells were split into 4x T175 flasks containing at least 20 million cells each and grown for 48 hours prior to treatment. Cells were treated every 48 hours with either 7.5x IC_50_ of 3′-dA or 7x, 14x, and 18x IC_50_ of NUC-7738. Cell media was removed and replaced with new media containing 3′-dA or NUC-7738. After 12 days, cell culture media was removed and replaced with new media without drugs. After selection, resistant colonies were expanded to approximately 3 × 10^7^ cells and used for genomic DNA isolation and insertion-site retrieval. Gene-trap insertion sites were recovered by linear amplification of genomic DNA sequences flanking the retroviral integration sites, sequenced, mapped to the human genome (hg19), and analyzed for enrichment of disruptive insertions over the control data set as described before (32).

### RNA-seq and analysis

Cells were treated for 6 hours with 46 μmol/L (IC_50_) and 375 μmol/L (IC_90_) 3′-dA and 7.6 μmol/L and 125 μmol/L NUC-7738. Cells were washed with PBS and RNA was extracted using Quick RNA extraction kit with DNAse (Zymo Research) on column treatment and final EtOH precipitation step. RNA quality was assessed by RNA integrity number factor determination using Agilent RNA 6000 Nano kit on a Bioanalyzer. Libraries were prepared using Illumina TrueSeq stranded total RNA protocol with insert size 150 to 250 nt, multiplexed, and sequenced on an Illumina HISeq4000 system as 75 pair-end resulting in approximately 50 million reads per sample. Resulting reads were aligned to the human reference genome (GRCh37) using HiSAT2(v2.1.1). RNA-seq quality was assessed using RNA-SeQC and duplicate reads were removed using Picard MarkDuplicates tool (RRID:SCR_006525, Broad Institute). Mapped, deduplicated reads were counted using featureCounts (v1.5.0). Counted reads were analyzed for differential expression using R-studio (v3.4.3) with DESeq2 package (RRID:SCR_000154, v1.18.1). The HALLMARK and REACTOME pathway enrichment analyses (RRID:SCR_003485) were performed with Gene Set Enrichment Analysis (GSEA) software from Broad Institute using default settings. Leading edge analysis was performed with gene sets showing FDR < 0.25. Leading edge genes were extracted and the fold change of significantly differentially expressed genes was plotted as a heatmap using Graphpad prism (v8.3.0).

RNA from patient biopsy was extracted and sequenced as follows. Hematoxylin and eosin–stained formalin-fixed, paraffin-embedded (FFPE) sections were analyzed for percentage tumor area. Extraction of the FFPE tissues was performed using the Qiagen FFPE RNeasy kit. RNA was isolated from the macro-dissected tumor area; upon completion of the extraction, the RNA samples underwent quantity and quality assessment. cDNA libraries were prepared using the Roche KAPA RNA HyperPrep with RiboErase library preparation kit and sequenced on a NovaSeq 6000 as 76 pair-end resulting in approximately 100 million reads per sample. Raw sequencing data in FASTQ format were assessed using FastQC (RRID:SCR_014583). Reads were aligned to the human reference genome (GRCh38) using HiSAT2(v2.1.1), and duplicate reads were removed using Picard MarkDuplicates tool (Broad Institute). Mapped, deduplicated reads were then counted using Salmon (v1.4.0) using ‘quant’ function in alignment-based mode. Raw count data were then imported to TCC-GUI, an R-shiny graphical user interface application, and analyzed using edgeR (RRID:SCR_012802) for differential gene expression.

### Target gene knockdown and depletion

Cells were transfected with stealth siRNA (catalog no. HSS104782, catalog no. HSS104784, catalog no. HSS179235; ThermoFisher) targeting HINT-1 using Lipofectamine RNAiMAX (Invitrogen) following the manufacturer's protocol. At 48 hours posttransfection, cells were treated and cell viability was measured 96 hours posttransfection as described above. Samples for Western blot analysis were harvested 48 hours posttransfection. For gene-depletion experiments, approximately 4 × 10^5^ HAP1 cells were seeded per well in a 6-well plate and grown to approximately 50% confluency. After 24 hours, cells were transfected with lentiCRISPR v2 plasmid (GenScript) containing a guide RNA (gRNA) targeting gene of interest using Turbofectin (catalog no. TF81001, OriGene) according to manufacturer's instruction. The following sequences were used: HINT-1 CRISPR gRNA 2 (CCTGGTGGCGACACGATCTT) and NUDT2 CRISPR gRNA 2 (AATTGCATTGTTGTCCACTT). At 24 hours posttransfection, positively transfected cells were selected using 2 μg/μL puromycin for 72 hours. After selection, surviving colonies were expanded and used for downstream analysis. For clonal selection, clones were selected using limited dilution and expanded into a 6-well plate for Western blot screening.

### Colony formation assay

HAP1 cells were seeded at a density of 10,000 cells per well in 6-well plates and indicated drug concentrations were added. Media was replaced every 48 hours until all cells from the control plate died. Cells were further grown in normal media until colonies were visible. Cells were washed twice with PBS and stained with 0.5% crystal violet in 20% methanol for 20 minutes.

### Cell lysis, fractionation, and Western blotting

Cells were harvested and lyzed using RIPA lysis and extraction buffer (Thermo Scientific) following manufacturer's protocol. Twenty to 30 μg of total protein lysates was loaded on Mini-PROTEAN TGX precast gels (Bio-Rad). Separated proteins were transferred to a polyvinylidene difluoride (PVDF) membrane using Trans-Blot Turbo Transfer System (Bio-Rad) followed by incubation with primary and horseradish peroxidase (HRP)–conjugated secondary antibodies [anti-mouse, catalog no. 7076S, Cell Signaling Technology (CST) or anti-rabbit, catalog no. 7074S, CST] and ECL Prime Western blotting (GE Healthcare) detection. For fractionation, cells were grown in 10-cm tissue culture plates and treated with 30 or 60 μmol/L of 3′-dA or NUC-7738 for 6 hours. Cells were grown in 10-cm tissue culture plates and fractionated using the Nuclear Extraction Kit (Abcam) following manufacturer's instructions. Twenty-five micrograms of each fraction was used for Western blot (WB) detection. NF-κB p65 antibody (catalog no. 8242, CST), nuclear marker Lamin B1 (catalog no. ab16048, Abcam), cytosolic GAPDH (catalog no. 97166, CST), cleaved PARP (catalog no. 9541, CST), and Actin (MAB1501, Merck) were used.

### Preclinical toxicology

NUC-7738 was administered to beagle dogs in 4 weekly cycles and reversal of any observed effects were evaluated during a final 2-week treatment-free period. Four dosing groups were defined, each comprising 3 males and 3 females. Three groups received NUC-7738 at 5 mg/kg/day, 10 mg/kg/day, and 20 mg/kg/day, respectively, and the fourth received vehicle (polyethylene glycol 400:0.9% NaCl solution; 70:30 w/w). In all groups, dosing was once daily for 5 consecutive days followed by a 2-day treatment-free interval for a total of 4 weeks (i.e., 4 treatment cycles). The following endpoints/parameters were evaluated: body weight, food consumption, ophthalmoscopy, electrocardiography, clinical observations, haematology, coagulation, clinical chemistry, urine analysis, organ weights, gross pathology, and histopathology. Toxicokinetic (TK) parameters were evaluated on day 1 and at the end of the last dosing day (day 26) according to a serial profile. Concentrations of NUC-7738 and its metabolites (cordycepin and 3′-deoxyinosine) in plasma were determined using a validated bio-analytical LC-MS/MS method.

Preclinical toxicology studies were previously conducted by NuCana Plc and the data presented here were sourced from the toxicology study report (VPT5543). The dog toxicology study was reviewed and approved by a local ethical review board at the vendor that conducted the study.

### Tissue slice culture of clear cell renal carcinoma

A fresh sample of clear cell renal carcinoma (ccRCC) was placed in formalin to serve as a formalin control for our experiment. The sample was transported in precooled 199 medium (catalog no. 2154) supplemented with penicillin/streptomycin (1%), Normocin (0.2%), and Glutamax (2 mmol/L). The tissue sample was embedded in low-melting point agarose gel and sliced into 250-μm sections using a Vibratome VT1000S (Leica Biosystems). Tissue slices were put immediately into media (199 medium supplemented with 10% FBS, 2 mmol/L Glutamax, and 1% penicillin/streptomycin) containing different concentrations of NUC-7738 (0, 5, and 25 μmol/L) and incubated for 24 hours at 37°C. Treated tissue sections were then fixed using PFA for 1 hour at room temperature and stored in PBS at 4°C. The treated slices were later embedded in paraffin and subsequently sliced into 3-μm sections using a microtome. The sections were placed on slides and baked overnight at 40°C. Sections were dewaxed using xylene and rehydrated in a graded alcohol series followed by tap water. Slides were placed into hematoxylin for 5 minutes, washed with running tap water, and incubated in Scott tap water substitute for 1 minute until the tissue sections turned blue. Slides were then placed in eosin Y aqueous solution for 5 minutes, rinsed in tap water, dehydrated in a graded alcohol series, cleared with xylene, and mounted in dibutylphthalate polystyrene xylene (DPX).

### Immunofluorescence staining

Immunofluorescence staining was performed using BOND RX. NF-κB p65 (catalog no. 4764, CST) and cleaved caspase-3 (catalog no. 9661, CST) were detected with HRP-conjugated secondary antibody and visualized using tyramide-fluorophore conjugates (TSA Cy5 for both). Nuclear counterstain was done using Hoechst. Slides were then scanned using Zeiss Axio Scan Z1.

### IHC Staining

Coverslips were lifted post multiplex staining (data not shown) and NF-kB (catalog no. 8242) and HINT1 (catalog no. HPA044577, Atlas Antibodies) antibodies were stained chromogenically on the Leica BOND autostainer. Antigen retrieval was performed at 100°C for 20 minutes with Epitope Retrieval Solution 1 (NF-κB) and 2 (HINT1), followed by primary antibody incubation at 1/800 for NF-κB and 1/500 for HINT1 dilution for 30 minutes then detection using the BOND Polymer Refine Detection System (DS9800, Leica Biosystems) as per manufacturer's instructions.

### IHC and analysis of HINT1 expression in tissue microarray

Three micron-thick FFPE sections from the tissue microarrays (TMA) were cut and incubated in a 60°C oven overnight. The sections were deparaffinized in xylene and rehydrated in gradually concentrated alcohols (100%, 100%, 80%, and 50%) and tap water. The epitopes of antigens on FFPE tissue were unmasked in 0.1 mol/L sodium citrated buffer using electric pressure cooker for 5 minutes, followed by 3% hydrogen peroxide (Sigma, catalog no. H1009) and serum-free protein block (Agilent, catalog no. X090930–2) for 5 minutes and 10 minutes, respectively. The sections were incubated with anti-HINT1 antibody (Abcam, catalog no. ab124912, 1:1,500 dilution) for 1 hour at room temperature, followed by 30 minutes of HRP-conjugated anti-rabbit secondary antibody (Agilent, catalog no. K346811–2) and were visualized by 3,3′-diaminobenzidine (DAB) chromogen (Agilent, catalog no. NEL745B001KT) for 10 minutes. Then the sections were counterstained with hematoxylin for 1 minute, followed by bluing agent and Scott tap water substitute for 1 minute. The sections were dehydrated in gradually concentrated alcohols (50%, 80%, 100%, and 100%) and xylene and mounted in DPX mountant (Cellpath, catalog no. SEA-1304–00A).

The Allred score, a combination of proportion of positive cells and the predominant intensity of the protein expression (33), was assessed by two observers (I.H. Um and D.J. Harrison).

### Clinical study

A Safety Study of NUC-7738 in Patients with Advanced Solid Tumours or Lymphoma, ClinicalTrials number: NCT03829254. Approved by Oxford C Ethics Committee ref 18/SC/0628.

### Data availability

The toxicologic data analyzed in this study are available from NuCana plc. Restrictions apply to the availability of these data, which were used under license for this study. All raw sequencing data are available from the authors upon reasonable request with the permission of NuCana plc. Other data generated in this study are available within the article and its supplementary data files.

## Results

### Chemical synthesis of ProTide form of 3′-dA: NUC-7738

**Figure 1.**
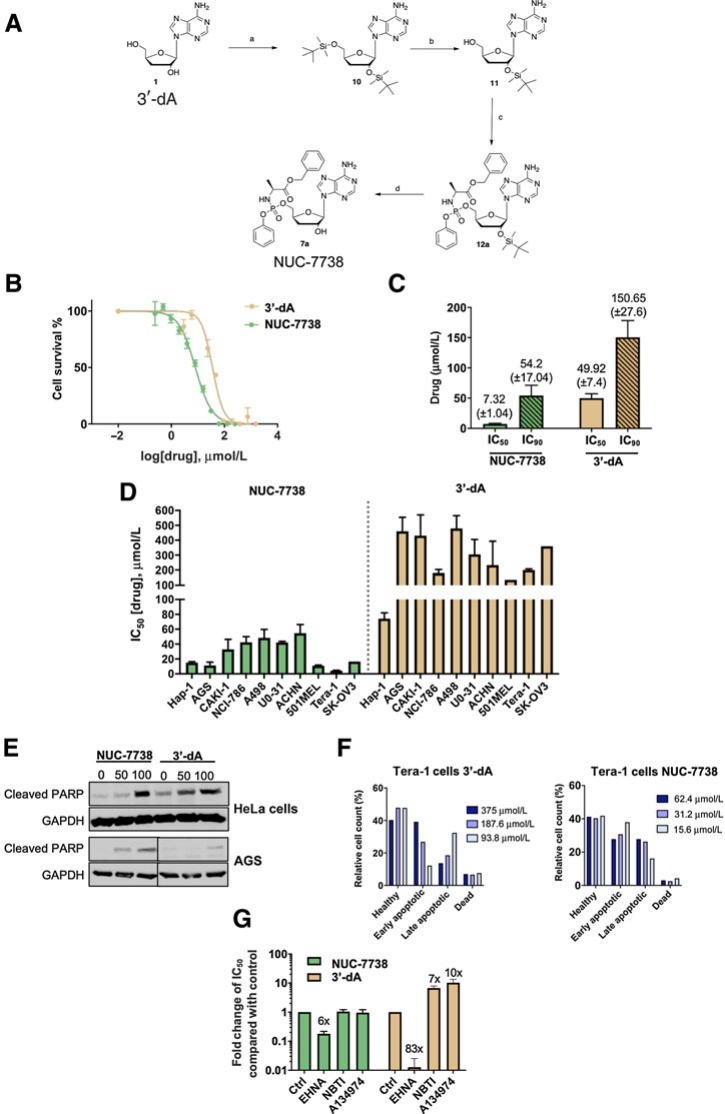
NUC-7738, ProTide version 3′-dA, has cytotoxic activity. **A,** Scheme of the chemical synthesis of 3′-deoxyadenosine 5′-O-phenyl-(benzyloxy-L-alaninyl)-phosphate (NUC-7738), a functionalized ProTide of 3′-dA. **B,** HAP1 cells were treated with different concentrations of 3′-dA or NUC-7738 and cell viability was measured after 48 hours. One representative plot is shown. **C,** IC_50_ and IC_90_ determination of 3′-dA or NUC-7738 was performed using a nonlinear regression model. Mean and SEM of 4 biological replicates are shown. **D,** Determination of IC_50_ of 3′-dA or NUC-7738 in gastric, renal, melanoma, and ovarian cancer cell lines. Mean and SEM of at least 3 biological replicates is shown (where no SEM is shown, only 2 biological replicates were performed). **E,** Gastric or cervical cancer cells were treated with NUC-7738 or 3′-dA for 24 hours, following which cleaved PARP was detected by Western blot. GAPDH was used as a loading control. **F,** Tera-1 cells were treated with NUC-7738 or 3′-dA for 24 hours. Early and late apoptotic and dead cells were sorted and counted after staining with Guava Nexin kit. **G,** Chemical perturbation of known enzymes involved in processing of 3′-dA. HAP1 cells were treated with NUC-7738 and 3′-dA in the presence or absence of the ADA antagonist EHNA, the hENT1 antagonist NBTI, or the ADK inhibitor A134974. The fold-change difference in IC_50_ values compared with control is shown as means with SEM (*n* > 3).

A preactivated or monophosphorylated version of 3′-dA [3′-dAMP or cordycepin monophosphate (CMP)] was synthesized using ProTide chemistry in a process similar to that described previously (28). This generated several preactivated 3′-dA prodrugs. Of these, 3′-deoxyadenosine-5′-O-phenyl-(benzyloxy-L-alaninyl)-phosphate or NUC-7738 (Fig. 1A) was selected for further evaluation. A protection–deprotection method described in Materials and Methods was used to prepare NUC-7738 with an overall yield of 42% and a purity of more than 99%.

### Pharmacologic comparison of NUC-7738 with 3′-dA

The potency of NUC-7738 was compared with its parent compound 3′-dA by measuring mean half-maximal inhibitory concentrations (IC_50_) across a wide set of cancer cell lines (Fig. 1B–D). NUC-7738 revealed greater potency, with a mean IC_50_ of 18.8 μmol/L, compared with 137.8 μmol/L for 3′-dA (Fig. 1D and Supplementary Table S1), particularly in certain lines such as teratocarcinoma (Tera-1) which were more than 40-fold more sensitive to NUC-7738. Both NUC-7738 and 3′-dA induced apoptotic cell death, shown by detection of cleaved PARP-1, in cells treated with either 3′-dA or NUC-7738 at 50 and 100 μmol/L (Fig. 1E and F).

To assess whether NUC-7738 was resistant to ADA and could bypass ADK and hENT1 (SLC29A1), cell viability was measured after treatment with their specific and corresponding protein inhibitors: *erythro*-9-(2-Hydroxy-3-nonyl) adenine hydrochloride (EHNA), A134974, and S-(4-nitrobenzyl)-6-thioinosine (NBTI), respectively (16, 17, 34, 35). Whilst 3′-dA was dependent on ADA, hENT1, and ADK as expected (Fig. 1G), the potency of NUC-7738 was mostly unaffected by inhibition of ADA or by inhibition of hENT1 or ADK.

### Genome-wide haploid genetic screen identifies genes that confer resistance to 3′-dA and/or NUC-7738

**Figure 2.**
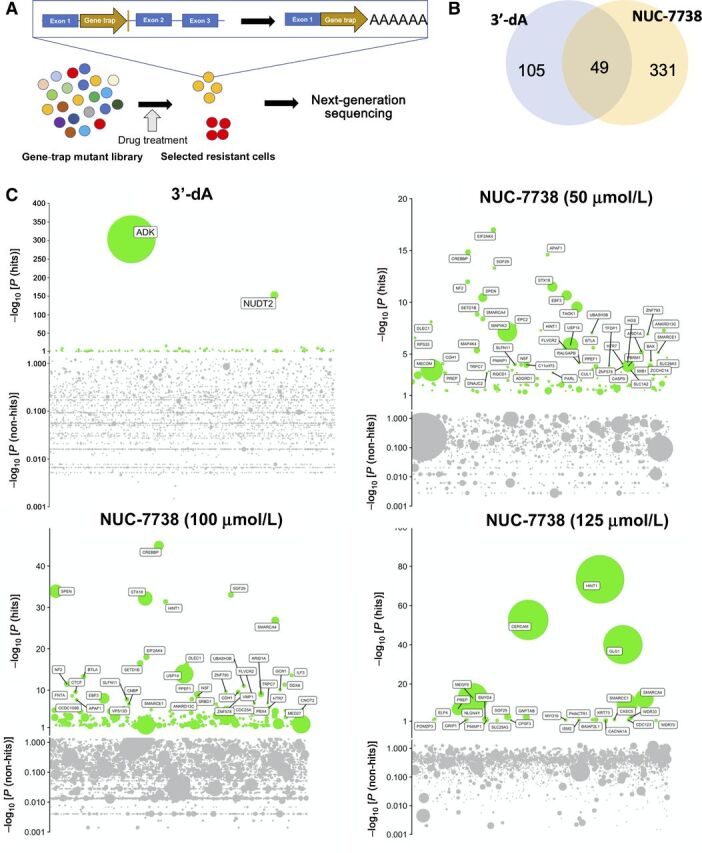
Genome-wide haploid genetic screen identifies genes necessary for the activity of 3′-dA and NUC-7738. **A,** Flowchart of insertional mutagenesis haploid screen. **B,** Venn diagram illustrating the overlap of genes found in 3′-dA and NUC-7738–treated samples. **C,** Bubble plots of 4 independent experiments. 3′-dA and NUC-7738 treatment was performed at given concentrations. *P* value is given on Y axis and genes sorted by their genomic location on X axis. Bubble sizes represent the number of unique insertions that were detected.

To identify genes which, when activated, confer resistance to 3′-dA and NUC-7738, we performed an insertional mutagenesis screen using the near-haploid human cell line HAP1 (Fig. 2 and Supplementary Fig. S1; refs. 29–32). In short, approximately 20 million HAP1 cells were mutagenized using a retroviral gene-trap vector as described (31), expanded ten-fold, and treated with a high concentration (≥7x IC_50_) of 3′-dA or NUC-7738. After 12 days, drug was washed off and resistant cells were expanded and harvested. The insertion sites of the gene trap were recovered by linear-amplification mediated–polymerase chain reaction (LAM-PCR) and Illumina sequencing. As a control, we sequenced the insertions of mutagenized cells that were expanded for the same period of time but not exposed to a drug.

Drug-treated cells contained insertions in fewer genes than in the unselected controls. As expected, insertions in *ADK* were strongly enriched in 3′-dA–treated cells (Fig. 2; Supplementary Tables S2 and S3). We subsequently validated a selection of genes using CRISPR/Cas9-mediated gene deletion: *HINT1, NUDT2, ADK, SMARCA4, UBE2G1*, and *CREBBP* (Supplementary Fig. S2). Besides *ADK*, the strongest insert enrichment with 3′-dA treatment was found in diadenosine tetraphosphatase (*NUDT2*). Specific resistance to 3′-dA, but not to NUC-7738, was confirmed (Supplementary Fig. S2B and S2C). Several other enriched genes were confirmed in follow-up experiments including *SMARCA4, UBE2G1*, and *CREBBP*, which contribute to 3′-dA sensitivity by a yet to be determined mechanism (Supplementary Fig. S2A). Of note, as expected, we did not see an enrichment for *ADA*, since depletion of *ADA* renders HAP1 cells hypersensitive to 3′-dA.

**Figure 3.**
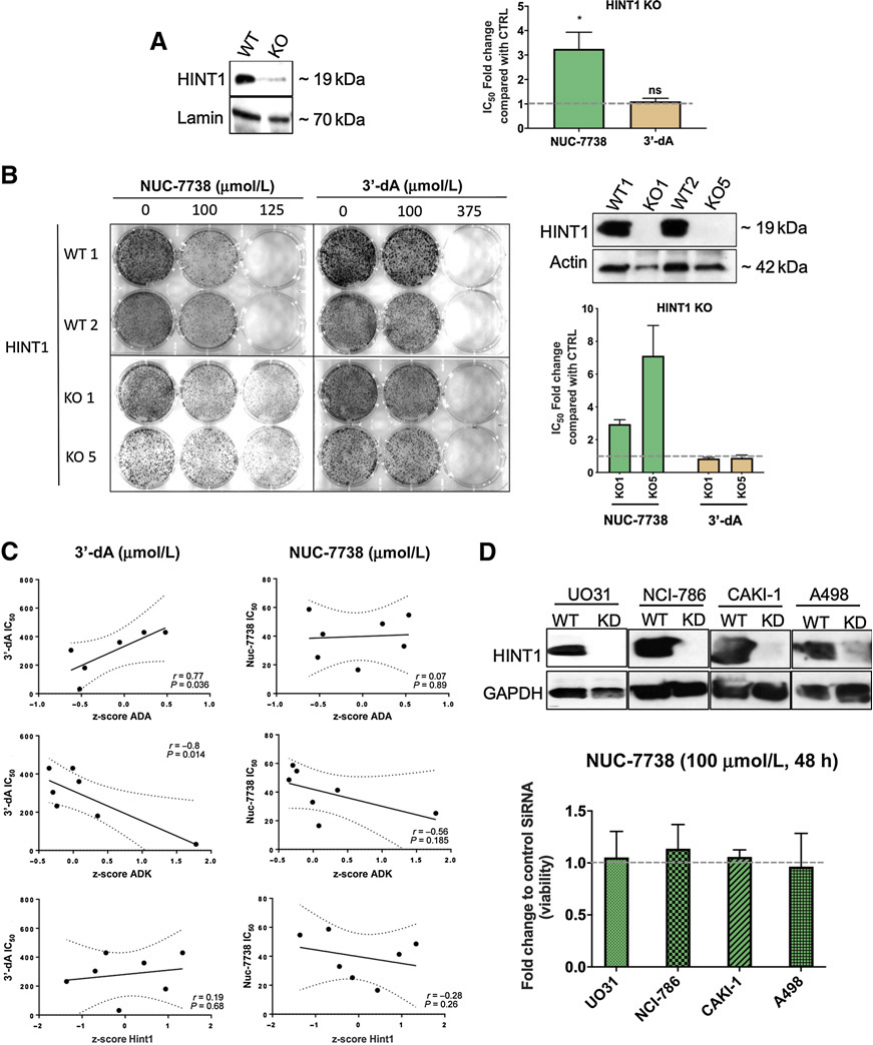
Validation of top hits from genome-wide haploid screen. **A,** HINT1 was deleted in HAP1 cells using CRISPR/Cas9 technology, as shown by Western blot analysis of HINT1 KO cells using specific antibodies to HINT1. WT and KO cells were treated with either NUC-7738 or 3′-dA. IC_50_ values were determined and the fold change between KO and WT cells was calculated. **B,** Single-cell–derived clonal knockouts for HINT1 (KO1 and KO5) and 2 WT isogenic cell lines. Western blot analysis of HINT1 KO cells using specific antibodies to HINT1 is shown. Following treatment with NUC-7738 or 3′-dA, IC_50_ values were determined and the fold change between KO and WT cells were calculated. **C,** Correlation between IC_50_ of NUC-7738 or 3′-dA and mRNA abundance in different NCI-60 cells is given for ADA, ADK, and HINT1. mRNA expression levels are given as z-score calculated across all NCI-60 cell lines. mRNA expression levels were obtained from CellMiner^TM^ v2.4.2. **D,** Western blot validation of siRNA-mediated HINT1 knockdown in renal cancer cell lines. WT and knockdown cells were treated with either NUC-7738 or 3′-dA. Graph shows the fold change in percentage of viable cells normalized to control (*n* = 3) after treatment with NUC-7738.

We performed the screen with NUC-7738 at 3 different concentrations (50, 100, and 125 μmol/L). In contrast to the screen with 3′-dA, none yielded an enrichment of *ADK* (Supplementary Table S2), confirming a lack of dependence on this gene (Fig. 1G). Surprisingly, the phosphoramidase *HINT1*, a gene known to be involved in purine metabolism, was strongly enriched in all three screens. Depletion of *HINT1* by CRISPR/Cas9 in HAP-1 cell lines and in single cell-derived clonal HINT1 knockout (KO) cells markedly reduced sensitivity to NUC-7738 but not 3′-dA (Fig. 3A and B). As expected, HINT1-depleted clonal KO cells were capable of forming colonies even after exposure to high concentrations of NUC-7738, but not after treatment with 3′-dA. Additionally, NUC-7738–induced apoptosis was noticeably attenuated upon HINT1 deletion (Supplementary Fig. S2D).

To explore the impact of HINT1 on sensitivity to NUC-7738, we grouped cancer cell lines into low, medium, and high according to their relative HINT1 mRNA expression levels and treated them with NUC-7738 or 3′-dA (Supplementary Fig. S2E). We observed no correlation between expression of HINT1 and sensitivity to NUC-7738 (r = 0.28, *P* = 0.26) or 3′-dA (r = 0.19, *P* = 0.68; Fig. 3C). We then depleted HINT1 in cell lines until only minimal residual levels were detectable on Western blotting and again observed no difference in sensitivity to NUC-7738 (Fig. 3D). By IHC we observed ubiquitous expression of HINT1 in most of the tested cancer tissues (Supplementary Fig. S3), which is in line with the expression pattern reported in the protein atlas (https://www.proteinatlas.org). These findings indicate that even low levels of HINT1 are sufficient for the activation of NUC-7738.

### Core target genes underpin the activity of both 3′-dA and NUC-7738

**Figure 4.**
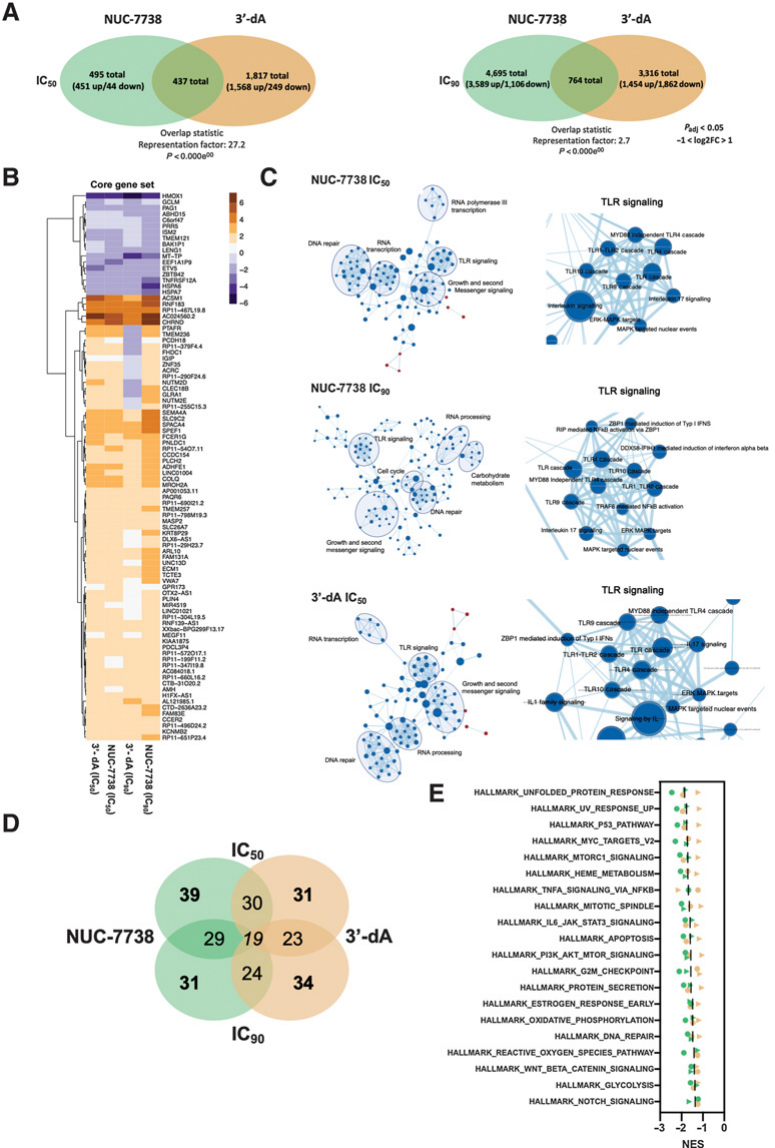
Transcription profiling of HAP1 cells treated with 3′-dA and NUC-7738. **A,** Venn diagram summarizing the number of differentially expressed genes (*P*_adj_ < 0.05 and −2>FC>2) in 3′-dA and NUC-7738–treated samples. **B,** Expression heatmap of 91 genes commonly differentially expressed in all 4 tested conditions (*P*_adj_ < 0.05 and −2>FC>2). GSEA and network mapping using Cytoscape. Genes ranked according to their *P* value and GSEA were performed on REACTOME gene sets. Gene overlaps between different pathways are shown. Nodes are GSEA enriched pathways while Edges represent overlapping shared genes between two pathways. **C,** GSEA and network mapping using Cytoscape. Genes ranked according to their *P* value and GSEA was performed on REACTOME gene sets. Gene overlaps between different pathways are shown. Nodes are GSEA enriched pathways while Edges represent overlapping shared genes between two pathways. **D,** Venn diagram summarizing number of overlapping enriched pathways by GSEA using Molecular Signature Database collection of HALLMARK gene. **E,** Summary of top 20 enriched pathways for Hallmark set enrichment analysis of hits for NUC-7738 and 3′-dA–treated cells. Green and orange indicate NUC-7738 and 3′-dA, respectively. Circles and triangles indicate dosing at IC_50_ and IC_90_, respectively.

To compare the impact of 3′-dA and NUC-7738 on transcription, we performed RNA-seq on cells after treatment at the relative IC_50_ and IC_90_ concentration for each compound (Fig. 4). A principal component analysis showed clear separation between treated and nontreated samples and a clear distinction in gene expression between them (Supplementary Fig. S4A–C). Whilst 3′-dA treatment induced minimal [fold change (FC) < 2] but significant changes in multiple genes, NUC-7738 caused greater change to a smaller number of genes (FC > 2). There was significant overlap of 91 genes that were differentially expressed by more than two-fold at all four conditions, forming a core set of NUC-7738 and 3′-dA–affected genes (Fig. 4B). Of note, NUC-7738 and 3′-dA had greatest impact on the expression of coding mRNAs, whilst less than 30% were noncoding or pseudogenes (Supplementary Fig. S4D).

### Both NUC-7738 and 3′-dA downregulate genes involved in cell survival

To understand whether genes of certain pathways were affected by drug treatment, we conducted pathway and GSEA (Fig. 4C–E). In general, we only observed gene set enrichments in downregulated genes, apart from the coagulation pathway which was upregulated after both treatments (Supplementary Fig. S4E). Amongst the downregulated transcripts, we found 19 pathways that were enriched after both 3′-dA and NUC-7738 treatment (Fig. 4D). These transcripts are involved in cell-growth signaling (PI3K-AKT-MTOR, NOTCH, or WNT-Beta-Catenin Signaling), cell division (Mitotic Spindle, G2M Checkpoint), and DNA repair (UV-response_U or DNA Repair; Fig. 4E).

### NUC-7738 inhibits the nuclear translocation of NF-**κ**B p65

**Figure 5.**
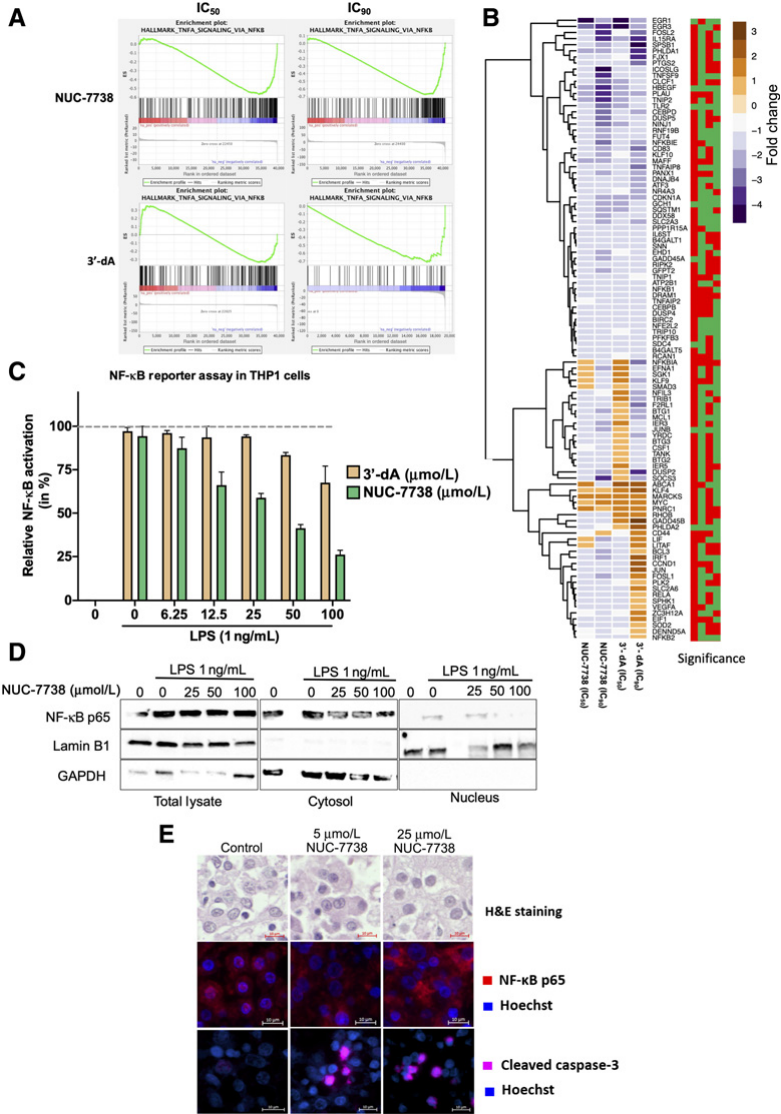
NUC-7738 and 3′-dA affect the NF-κB pathway. **A,** Enrichment plots for NF-κB pathway for all 4 conditions. ES, Enrichment score. **B,** Expression levels of genes found in the leading edge of NF-κB pathway enrichment. Blue indicates downregulation and brown indicates upregulation of transcript. Significance plot indicates whether the gene was significantly differentially expressed. Red stands for *P*_adj_ > 0.05 and green *P*_adj_ < 0.05. Order of columns corresponds with expression heatmap. **C,** NF-κB activity was measured using the SEAP reporter gene assay in THP-1 cells. NF-κB activity was induced with lipopolysaccharide (LPS) in the presence or absence of NUC-7738. SEAP production as result of NF-κB activity was measured using the QuantiBlue colorimetric enzyme assay. Values were normalized to untreated LPS stimulated cells. **D,** NF-κB activity was induced with LPS in the presence or absence of NUC-7738 in NF-κB THP1 reporter cell line. Cells were harvested and fractionated into nuclear and cytosolic fraction. Western blot analysis using specific antibodies was employed to detect NF-κB p65 (RELA), nuclear marker Lamin B1, and cytosolic marker GAPDH. **E,** NF-κB p65 and Caspase 3 in *ex vivo* tissue treated with NUC-7738 for 24 hours. NF-κB p65 was seen in the nucleus of controls but disappeared after treatment with NUC-7738, and an increase in Caspase 3 was observed after 24 hours. H&E, Hematoxylin and eosin.

Interestingly, amongst affected pathways, the NF-κB signaling cascade was one of the most strongly enriched after 3′-dA and NUC-7738 treatment (Fig. 5A and B). NF-κB builds a critical link between inflammation and cancer progression, which makes it an attractive therapeutic target (36–38). To validate the impact of 3′-dA and NUC-7738 on NF-κB signaling, cells were stably transfected with a NF-κB reporter construct and activation of NF-κB was calorimetrically quantified. NUC-7738 and, to a lesser extent, 3′-dA treatment significantly reduced NF-κB activation (Fig. 5C). Using a NF-κB reporter cell line, a reduction in the nuclear translocation of NF-κB was also observed after treatment with NUC-7738 (Fig. 5D). Similar results were also obtained in the NUC-7738–treated renal cancer cell lines 786-O (IC_50_ = 13 μmol/L) and UM-RC-2 (IC_50_ = 4 μmol/L) when fluorescence stained for intracellular RELA (p65; Supplementary Fig. S5A). To further validate this observation in tumor cells, we treated tissue slices of freshly obtained human ccRCC with increasing concentrations of NUC-7738 and observed a dose-dependent decrease in nuclear p65 and an increase in tumor-cell apoptosis (Fig. 5E and Supplementary Fig. S5B).

### Preclinical toxicity and toxicokinetics of NUC-7738

Owing to species-specific serum esterases which rapidly degrade the phosphoramidate moiety, NUC-7738 is unstable in rat and mouse serum, with a half-life of less than 2 minutes in the rat compared with 424 minutes in human (39, 40). Rodent models therefore cannot be used to assess toxicity and toxicokinetics of NUC-7738 and its metabolites (3′dA and 3′deoxyinosine). Instead, beagle dogs were administered NUC-7738 in dose-escalation cohorts. Each cohort received NUC-7738 by slow intravenous infusion followed by a 2-day break for 4 cycles in total. This was followed by a 2-week treatment-free period. Toxicokinetic parameters were evaluated on day 1 and at the end of the last dosing day (day 26) according to a serial profile. No significant differences (where significance is considered to be >2-fold change) in NUC-7738 systemic exposure (as mean C_max_ and AUC 0-t) were observed following single and repeated administrations at all doses tested. No significant gender differences in systemic exposure were observed. Exposure to metabolite 3′-deoxyinosine at all doses and on both occasions and genders, in terms of AUC 0-t, was generally comparable to that of NUC-7738 (mean ratio metabolite/parent was 1.32), whereas in terms of C_max_, metabolite exposure was consistently lower than parent (mean ratio metabolite/parent was 0.42). Median T_max_ for NUC-7738 and 3′-deoxyinosine occurred at 0.25 hours after the end of infusion. Mean plasma clearance (Cl) for NUC-7738 at all doses and on both TK occasions in both genders was in the range 14,700–32,000 ml/h/kg, with mean distribution volume at steady state (Vss) ranging from 7,320–21,100 ml/kg whilst the mean elimination half-life (t½) was between 0.22 and 0.46 hours. In conclusion, NUC-7738, when administered for a total of 4 cycles (4 weeks) at 5, 10, and 20 mg/kg/day, by intravenous slow injection, induced changes in the liver and bone marrow at 20 mg/kg/day. Based on the results of this study, the highest nonseverely toxic dose (HNSTD) was considered to be 10 mg/kg/day [mean AUC(0-t) 638 ng/h/mL and mean *C*_max_ 1,560 ng/mL for NUC-7738, based on sex mean at day 26 values].

### NUC-7738 is successfully metabolized to 3′-dATP in patients

**Figure 6.**
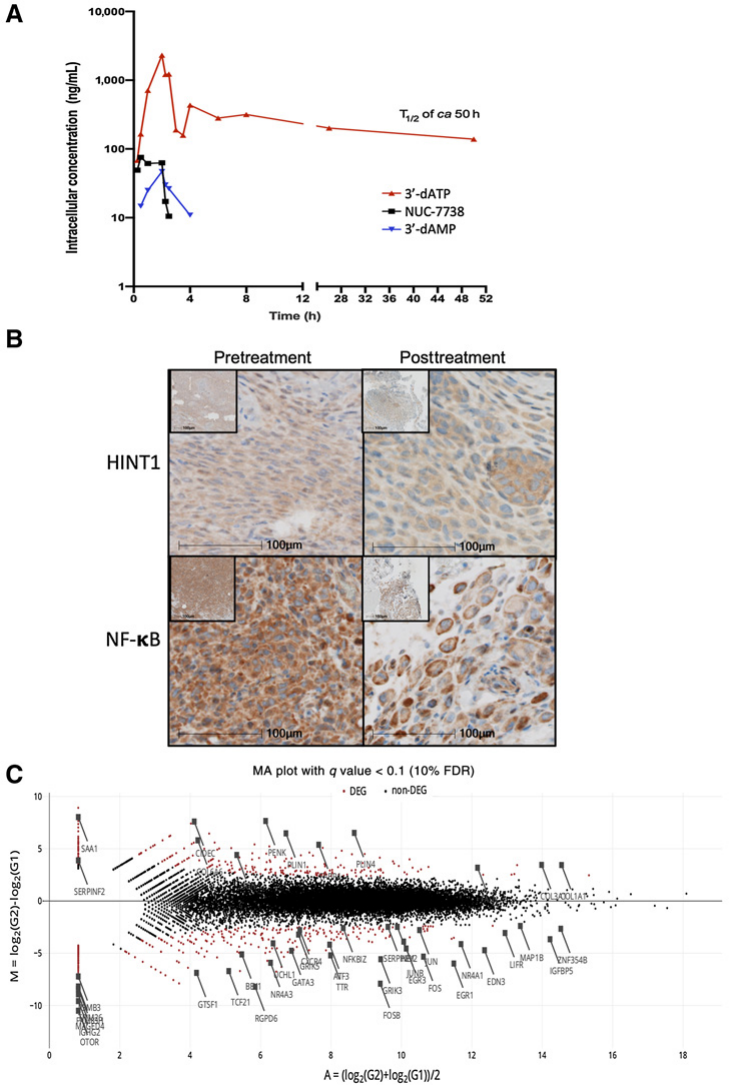
Clinical validation of the activity of NUC-7738. **A,** Intracellular levels of 3′-dATP, 3′-dAMP, and NUC-7738 were measured in the PBMCs from 7 patients who received treatment with NUC-7738 at a dose of 400–900 mg/m^2^ in the ongoing NuTide:701 clinical study. High intracellular levels of 3′-dATP were detected 0.25 hours after the start of infusion and were maintained for at least 48 hours. h, hours. **B,** IHC staining for HINT1 and NF-κB p65 subunit of pre- and posttreated tissue samples obtained from a melanoma cancer specimen. **C,** MA Plot summarizing transcriptomic profiling of pre- and posttreated tissue samples. Red dots indicate genes passing a FDR < 10%.

These data supported the initiation of NuTide:701, a first-in-human phase I dose-escalation/expansion study assessing the safety/tolerability, PK, and pharmacodynamics of NUC-7738 in patients with advanced solid tumors that were resistant to conventional treatment. This study is currently ongoing at three centers in the United Kingdom: Edinburgh, Newcastle, and Oxford. As of June 1, 2021, 28 patients with advanced cancers have been enrolled and received escalating doses of 14–900 mg/m^2^ (intravenous infusion from 30–120 minutes) once weekly until dose-limiting toxicity or disease progression occurs. Intrapatient dose escalation ensures those starting at lower dose levels of NUC-7738 are continuously moved to higher doses once safety has been established. The most common cancers to be included are immunotherapy-resistant melanoma (*n* = 8), colorectal cancer (*n* = 3), and gastric and lung cancer (*n* = 2 each). NUC-7738 has so far been well tolerated with no dose-limiting toxicities reported. Encouraging signals of antitumor activity and prolonged disease stabilization were observed, particularly amongst patients with immunotherapy-resistant melanoma (41). PK measurement of metabolites, in serum as well as peripheral blood mononuclear cells (PMBC) collected at regular timepoints after infusions, has revealed that NUC-7738 has a predictable plasma PK profile, with a dose-proportional increase in C_max_ and AUC. Across 7 patients treated with doses of NUC-7738 ranging from 400–900 mg/m^2^, high intracellular levels of 3′-dATP were detected in PBMCs 0.25 hours after the start of infusion and maintained for at least 48 hours, demonstrating successful uptake of NUC-7738 into PBMCs, release of the 3′-dAMP nucleotide, and efficient conversion to 3′-dATP (Fig. 6A). A posttreatment biopsy was taken from a patient with melanoma and compared with their archived specimen collected prior to enrolling in the clinical trial. The pre–NUC-7738 specimen shows presence of HINT1 and abundant cytoplasmic and nuclear NF-κB. The posttreatment sample shows some reduction in HINT1 and redistribution of p65 subunit of NF-κB to the cell periphery (Fig. 6B). Transcriptional profiling of the specimens using RNA-seq identified more than 1,069 genes which were at least 2-fold differentially expressed after treatment with NUC-7738 (Fig. 6C and Supplementary Fig. S6A–C). Interestingly, TNF-α signalling via the NF-κB pathway was significantly downregulated (Supplementary Fig. S6B) with a reduction ranging from 7- to 40-fold in expression of some of its prooncogenic target genes (e.g., *JUNB, FOS, DUSP1*) and melanoma-related genes (e.g., *EGR3, NR4A3, CCN1;* Supplementary Fig. S6D). Of note, our results in leukemia-derived HAP1 cells, acute monocytic leukemia–derived THP1 cells, and the human ccRCC *ex vivo* model overlap with those found in patient-derived melanoma specimens (Supplementary Fig. S6E), indicating that the effect on NF-κB signaling may be a universal feature of NUC-7738 treatment.

## Discussion

The natural nucleoside 3′-dA or cordycepin is an ancient medicine with a broad spectrum of purported health-related benefits and is widely used as a food supplement and herbal remedy. Studies conducted by ourselves and others have demonstrated that 3′-dA has potent anticancer effects but its half-life is limited to 1.6 minutes in plasma due to its rapid degradation by the ubiquitous enzyme ADA (42). For this reason, 3′-dA is not used as an anticancer therapy. Here we described the synthesis of NUC-7738, a new ProTide comprised of 3′-dAMP fused to a phosphoramidate (aryl, ester, and amino acid) moiety with the aim of overcoming these biochemical properties. As ProTide modification is designed to enhance stability and increase the intracellular delivery of the active nucleotide analogue, side-by-side comparisons were made between NUC-7738 and 3′-dA to determine proof-of-concept and to evaluate mechanisms of resistance and activation.

NUC-7738 was demonstrated to have 7- to 40-times greater potency than 3′-dA across a range of cancer cell lines and induced cell death by apoptosis. As expected, whereas 3′-dA was dependent on ADA, hENT, and ADK for its stability, uptake, and initial phosphorylation, NUC-7738 was resistant to breakdown by ADA, and did not require ADK for activation nor hENT1 for uptake. This supports the hypothesis that the phosphoramidate moiety protects d'-AMP from ADA-driven deamination, bypasses the initial ADK-dependent monophosphorylation step, and allows cell entry via an hENT-independent mechanism. No other transporter mutations were associated with decreased NUC-7738 uptake in our genetic screen, suggesting its uptake is not predominantly dependent on a single transporter. Here we show that the phosphoramidase HINT1 is required to activate NUC-7738 consistent with previous studies showing HINT1 can cleave the P-N bond of ProTides, a step required for the intracellular release of the active monophosphate (43). Importantly, HINT1 protein is present across numerous cancer types and its depletion was not dose-limiting, implying that even low levels of HINT1 are sufficient for NUC-7738 activation. In addition, we do not have any evidence that increased expression of HINT-1 correlates with increased toxicity in organs or tissue. This is in line with data from the clinical trial where we observed a predictable distribution of PK metabolites across all patients regardless of their tumor type and no difference between toxicity signals between them. Moreover, we have not yet observed side effects common to nucleoside analogues (including anemia, leukopenia, gastrointestinal, and thrombocytopenia) in study participants, suggesting that either normal tissue is less affected or that we are still outside the toxicity window in dosing terms.

NUC-7738 induced a cytotoxic effect on a wide range of cancer cell lines with an IC_50_ in the low μM range (<10 μmol/L). RNA-seq analysis revealed a transcriptome-wide downregulation of signaling pathways involved in cell survival and proliferation, particularly the NF-κB pathway. This cascade is active in many cancers, due to phosphorylation and inactivation of the NF-κB inhibitor IκBα, allowing the NF-κB p50-p65 heterodimer to enter the nucleus and drive transcription of proproliferative factors including TNF-α (38, 44, 45). Although rationally-designed NF-κB inhibitors have been developed, none have yet reached the clinic due to inefficacy or significant toxicity (36–38, 46, 47). In contrast, naturally-derived agents such as curcumin, resveratrol, and 3′-dA (48) have demonstrated NF-κB attenuation by blocking either the nuclear translocation of p65 or its DNA binding but invariably pose PK limitations due to their poor bioavailability. By applying ProTide chemistry to the 3′-dA, its bioavailability was enhanced whilst its native mechanism of action was retained. Subsequent and more detailed mechanistic studies, beyond the scope of this manuscript, will determine its exact target within the NF-κB cascade.

The results presented here, together with the encouraging PK data from the ongoing phase I trial, show that the ProTide NUC-7738 is resistant to ADA degradation and is capable of releasing active 3-dAMP into cells, where it is rapidly converted to the key anticancer metabolite 3′-dATP which was detected 2 hours after the start of infusion and has a half-life of approximately 50 hours. NUC-7738 has been well tolerated and has demonstrated encouraging signals of anticancer activity in patients with advanced solid tumors (49). These findings provide proof of concept that NUC-7738 overcomes the cancer resistance mechanisms that limit the activity of 3′-dA and support the further clinical evaluation of NUC-7738 as a novel cancer treatment within the growing pantheon of ProTides developed for the treatment of cancer.

## Authors' Disclosures

H. Schwenzer reports grants from NuCana PLC during the conduct of the study. M. Elshani reports other support from NuCana PLC during the conduct of the study. M. Kudsy reports grants from NuCana during the conduct of the study. V. Ferrari reports grants from NuCana PLC during the conduct of the study; in addition, V. Ferrari has a patent for WO 2016083830 A1 20160602 issued. S. Nijman reports other support from Scenic Biotech outside the submitted work. M. Serpi reports grants from Cardiff University during the conduct of the study. S. Symeonides reports grants and personal fees from MSD; grants from Verastem; personal fees from Ellipses, Medannex, Vaccitech, Eisai, Pfizer, and Merck Serono; and personal fees and non-financial support from BMS, EUSA, and Ipsen outside the submitted work. R. Plummer reports other support from NuCanca PLC during the conduct of the study, as well as personal fees from Pierre Fabre, Bayer, Novartis, Biosceptre, BMS, Cybrexa, Ellipses, CV6 Therapeutics, Astex Pharmaceuticals, Medivir, GammaDelta Therapeutics, Sanofi Aventis, AstraZeneca, and MSD outside the submitted work. D.J. Harrison reports grants and other support from NuCana PLC during the conduct of the study. S.P. Blagden reports grants from NuCana PLC during the conduct of the study; S.P. Blagden also reports other support from NuCana PLC, Redx, Sierra Oncology, Astex, Incyte, Tesaro, UCB, BerGenBio, and MSD, as well as personal fees from Ellipses, Amphista, and RAport outside the submitted work. No disclosures were reported by the other authors.

## Supplementary Material

Supplementary Figure 1 Figure S1 related to Figure 2: Genome wide haploid genetic screen identifies genes necessary for the activity of 3'-dA and NUC-7738. A) Number of unique gene trap sense insertions and significant gene hits found in the haploid genetic screen. B) Venn diagram indicating the overlap of significant hits found for NUC-7738 treatment.

Supplementary Figure 2 Figure S2 related to figure 3: Validation of top hits from genome wide haploid screen A) Genes of interest were deleted using an all-in-one gRNA-CRISPR/Cas9 construct and following Puromycin selection. Dose response curves of polyclonal HAP1 knockout cells (black) and wildtype control cells (red) from selected gene hits of the genome wide haploid screen. Cells were treated with NUC-7738 or 3'-dA for 48 hours. B) NUDT2 was deleted in HAP1 cells using all-in-one gRNA-CRISPR/CAS9, as shown by Western blot analysis of NUDT2 knockout cells using specific antibodies to NUDT2. Polyclonal wildtype control and knockout cells were treated with either NUC-7738 or 3'-dA. IC50 values were determined and the fold changes between knockout and wildtype cells were calculated. C) Single cell-derived clonal knockouts for NUDT2 (KO1 and KO2) and two wildtype isogenic cell lines. Western blot analysis of NUDT2 knockout cells using specific antibodies to NUDT2 is shown. IC50 values were determined following treatment with NUC-7738 or 3'-dA and the fold changes between knockout and wildtype cells were calculated. D) HINT1 knockout HAP1 cells were treated with 3'-dA or NUC-7738 and levels of cleaved PARP were analysed on a Western blot using specific antibodies.

Supplementary Figure 2E Figure S2E related to figure 3: Validation of top hits from genome wide haploid screen. E) mRNA expression levels are given as z-score calculated across all NCI-60 cell lines. mRNA expression levels were obtained from CellMinerTM v2.4.2.

Supplementary Figure 3 Figure S3 related to figure 4: HINT1 expression across selected cancer types. A) Examples histochemical staining of HINT1 in various types of cancer cells. B) Quantification of HINT1 expression in various types of cancer. The proportion of HINT1 positivity scored by the Allred score, which is combination of proportion of positive cells and the predominant intensity of the protein expression.

Supplementary Figure 4 Figure S4 related to figure 4: Transcriptional profiling of 3'-dA and NUC-7738 treated HAP1 cells. Principal component analysis from RNA sequencing to compare gene expression variance between NUC-7738 and 3'-dA treated cells versus control (A) and between the two drugs (B). C) Volcano plot analysis obtained from DEseq2 of NUC-7738 and 3'-dA treated cells at two different concentrations. Significant hits (padj&lt;0.05) are highlighted in purple. Non-significant hits are in red. Annotation of TOP20 hits according to adjusted p value are given. D) Relative proportion of protein coding and non-coding RNAs within the detected transcripts. E) Summary of enriched upregulated REACTOME pathways obtained from NUC-7738 and 3'-dA treated cells. Green and orange indicate NUC-7738 and 3'-dA, respectively. Circle and triangle represent dosing at IC50 and IC90, respectively.

Supplementary Figure 5 Figure S5 related to figure 5: NUC-7738 down-regulates cell survival pathways and induces apoptosis. A) A) Renal 786-O and UM-RC-2 cells were treated with NUC-7738 for 24 hours and stained for NF-kB p65 and nuclear dsDNA (DAPI). P65 was seen in the nucleus of controls but disappeared after treatment with NUC-7738. B) Haematoxylin and Eosin (H&E) staining of ex vivo tissue treated with NUC-7738 for 24 hours. H&E examination showed increased numbers of shrunken pyknotic nuclei corresponding to caspase 3 positive cells.

Supplementary Figure 6 Figure S6 related to figure 6: Transcriptomic profiling of post-treatment biopsy taken from one patient with melanoma. A) Violine plots of expression of genes pre- and post-treatment for all genes which showed, in at least one condition, more than 100 counts. B) Enrichment plot for TNF alpha signalling via NF-kB pathway (NES = -3.73, FDR=0). C) Expression of core gene set contributing to the leading edge of NF-kB pathway enrichment plot. D) Expressional changes pre-and post-treatment of TOP 10 genes extracted from NF-kB enrichment plot. E) Overlapping genes between all RNAseq data sets obtained during this study, stratified for NF-kB leading edge genes only.

Table S1 Table S1 related to Figure 1: NUC-7738 has cytotoxic activity

Table S2 Table S2 related to Figure 2: Genome wide haploid genetic screen identifies genes necessary for the activity of 3'-dA and NUC-7738

Table S3 Table S3 related to Figure 2: Genome wide haploid genetic screen identifies genes necessary for the activity of 3'-dA and NUC-7738.

Supplementary Materials NMR spectra for synthesised compounds
