# Supplementary figures and images for: The Novel Nucleoside Analogue ProTide NUC-7738 Overcomes Cancer Resistance Mechanisms In Vitro and in a First-In-Human Phase I Clinical Trial
Source: Clin Cancer Res. 2021 Dec 1;27(23):6500–13. doi: 10.1158/1078-0432.CCR-21-1652 (PMC9401491; doi:10.1158/1078-0432.CCR-21-1652)

Figure S1

A)

|                   | Unique<br>Insertions | Significant Hits |
|-------------------|----------------------|------------------|
| CONTROL           | 1 772 616            | ~16 200          |
| 3'-dA (375μM)     | 116 750              | 106              |
| NUC-7738 (50 μM)  | 809 901              | 121              |
| NUC-7738 (100 μM) | 326 843              | 295              |
| NUC-7738 (125 μM) | 5 160                | 27               |

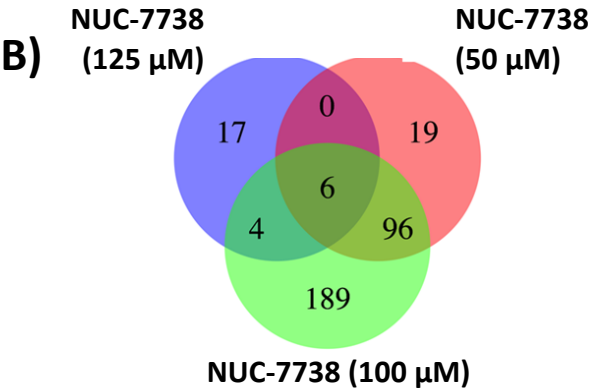

Supplement: Supplementary Figure 1 — Figure S1 related to Figure 2: Genome wide haploid genetic screen identifies genes necessary for the activity of 3'-dA and NUC-7738. A) Number of unique gene trap sense insertions and significant gene hits found in the haploid genetic screen. B) Venn diagram indicating the overlap of significant hits found for NUC-7738 treatment. [file 10780432ccr211652-sup-265190_3_supp_7351767_qyckvc.pdf]

# Figure S2

A)

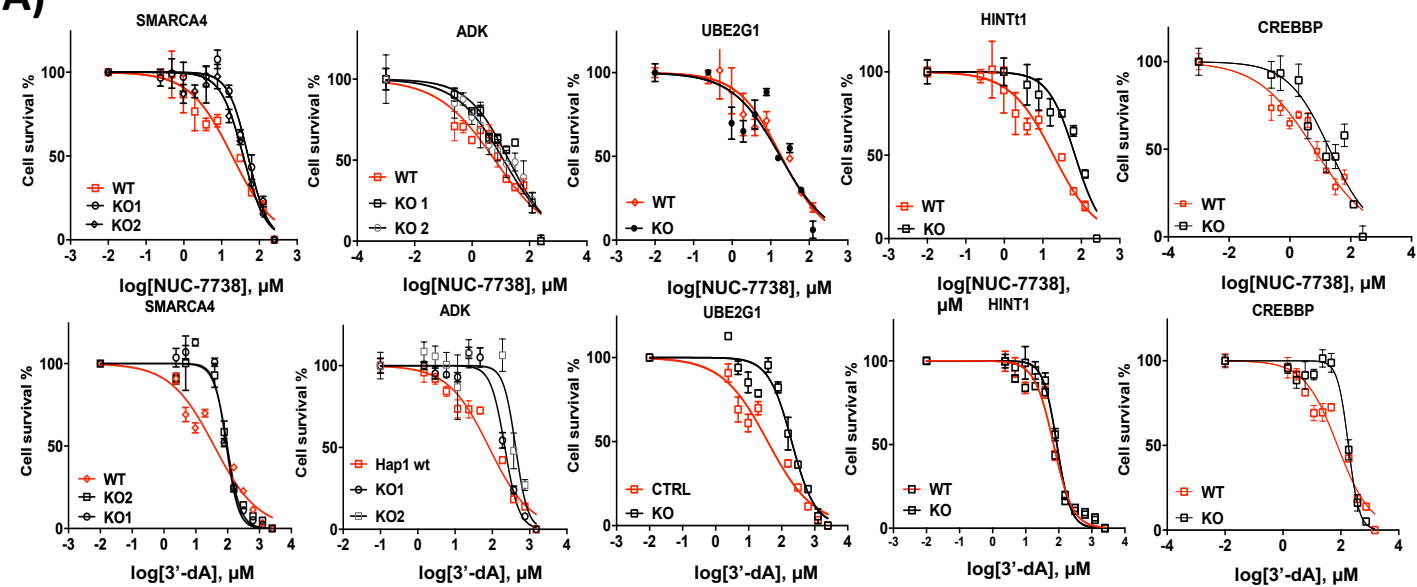

B)

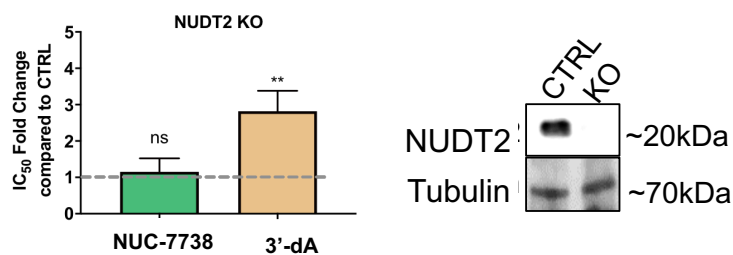

C)

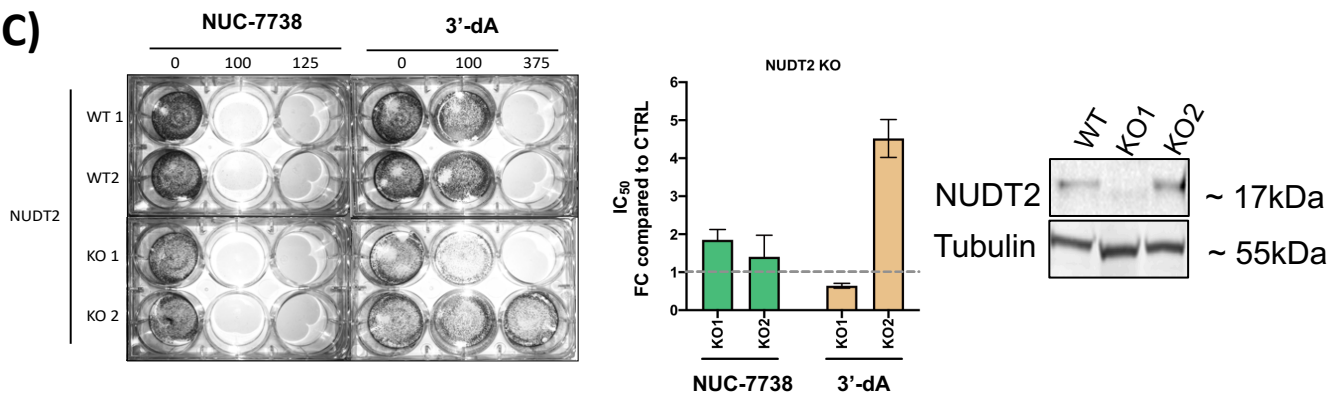

D)

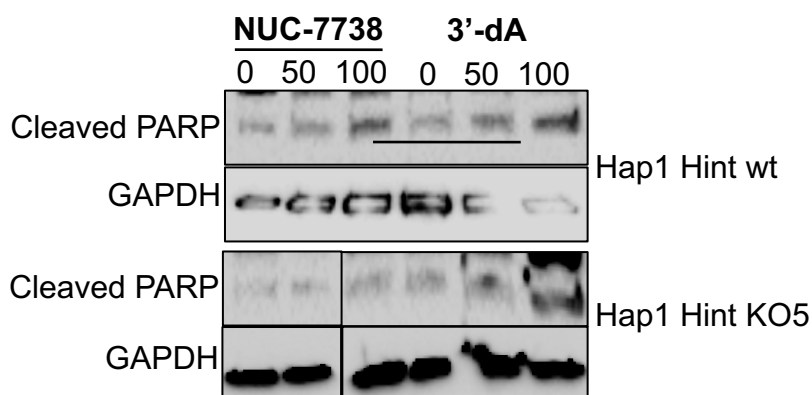

Supplement: Supplementary Figure 2 — Figure S2 related to figure 3: Validation of top hits from genome wide haploid screen A) Genes of interest were deleted using an all-in-one gRNA-CRISPR/Cas9 construct and following Puromycin selection. Dose response curves of polyclonal HAP1 knockout cells (black) and wildtype control cells (red) from selected gene hits of the genome wide haploid screen. Cells were treated with NUC-7738 or 3'-dA for 48 hours. B) NUDT2 was deleted in HAP1 cells using all-in-one gRNA-CRISPR/CAS9, as shown by Western blot analysis of NUDT2 knockout cells using specific antibodies to NUDT2. Polyclonal wildtype control and knockout cells were treated with either NUC-7738 or 3'-dA. IC50 values were determined and the fold changes between knockout and wildtype cells were calculated. C) Single cell-derived clonal knockouts for NUDT2 (KO1 and KO2) and two wildtype isogenic cell lines. Western blot analysis of NUDT2 knockout cells using specific antibodies to NUDT2 is shown. IC50 values were determined following treatment with NUC-7738 or 3'-dA and the fold changes between knockout and wildtype cells were calculated. D) HINT1 knockout HAP1 cells were treated with 3'-dA or NUC-7738 and levels of cleaved PARP were analysed on a Western blot using specific antibodies. [file 10780432ccr211652-sup-265190_3_supp_7351751_qymkvm.pdf]

Figure S2

E)

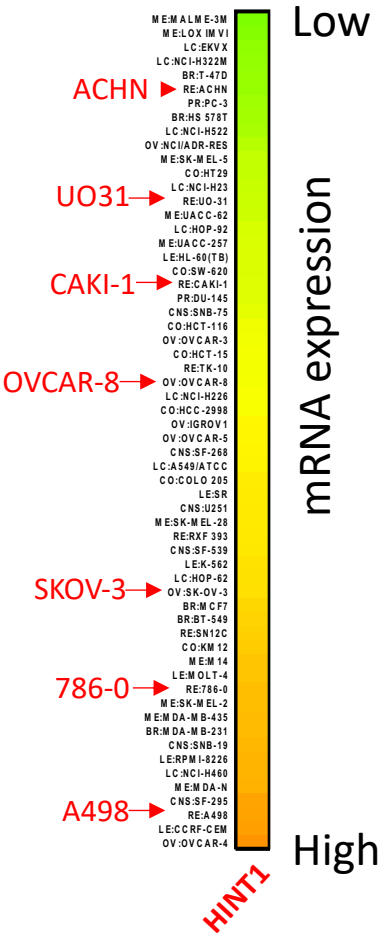

Supplement: Supplementary Figure 2E — Figure S2E related to figure 3: Validation of top hits from genome wide haploid screen. E) mRNA expression levels are given as z-score calculated across all NCI-60 cell lines. mRNA expression levels were obtained from CellMinerTM v2.4.2. [file 10780432ccr211652-sup-265190_3_supp_7351768_qyykvy.pdf]

Figure S3:

A)

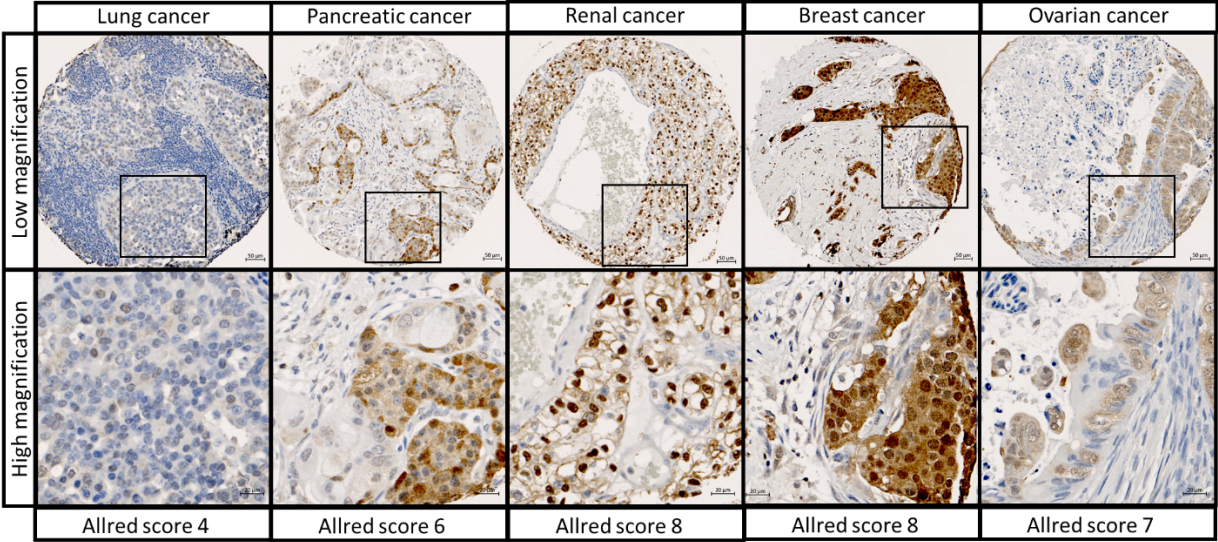

B)

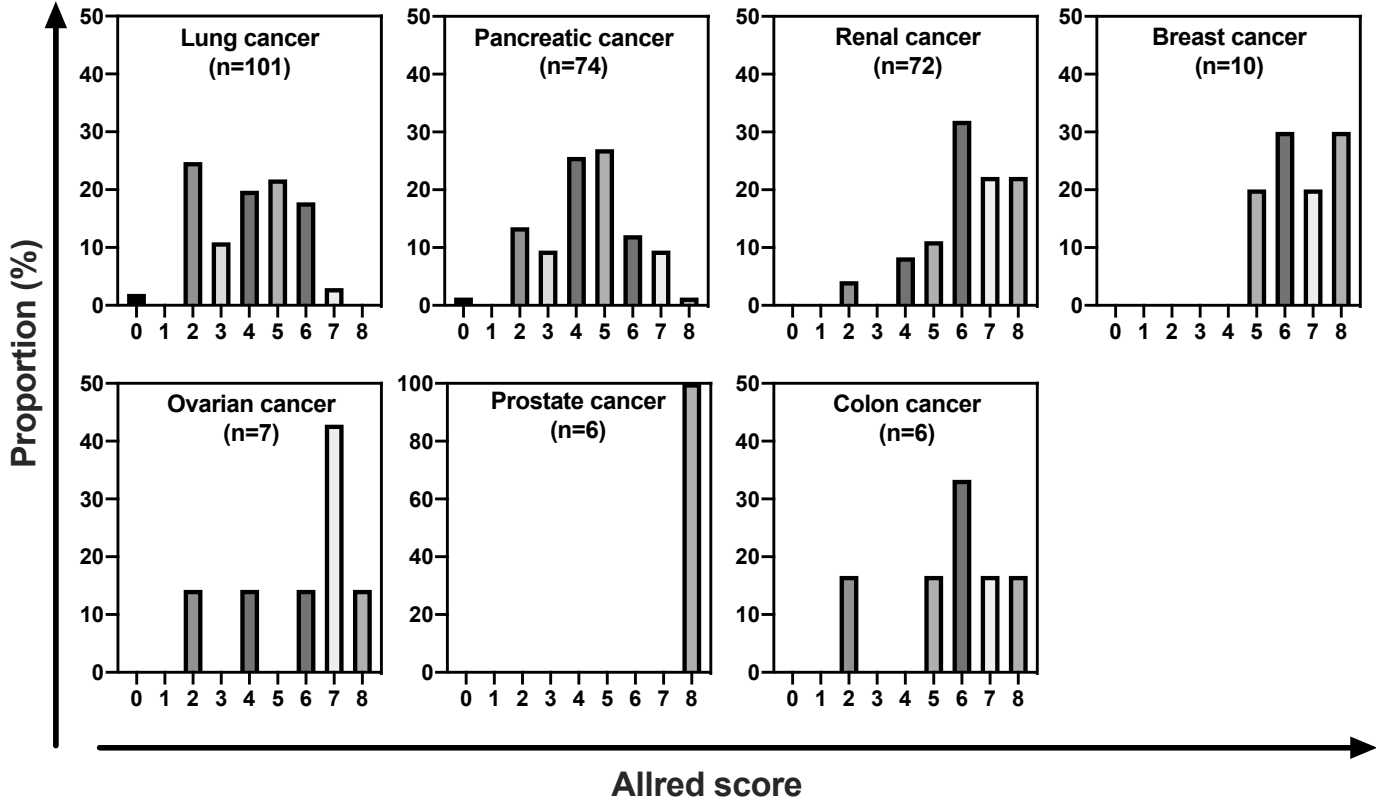

Supplement: Supplementary Figure 3 — Figure S3 related to figure 4: HINT1 expression across selected cancer types. A) Examples histochemical staining of HINT1 in various types of cancer cells. B) Quantification of HINT1 expression in various types of cancer. The proportion of HINT1 positivity scored by the Allred score, which is combination of proportion of positive cells and the predominant intensity of the protein expression. [file 10780432ccr211652-sup-265190_3_supp_7351753_qyqkvq.pdf]

## Figure S4

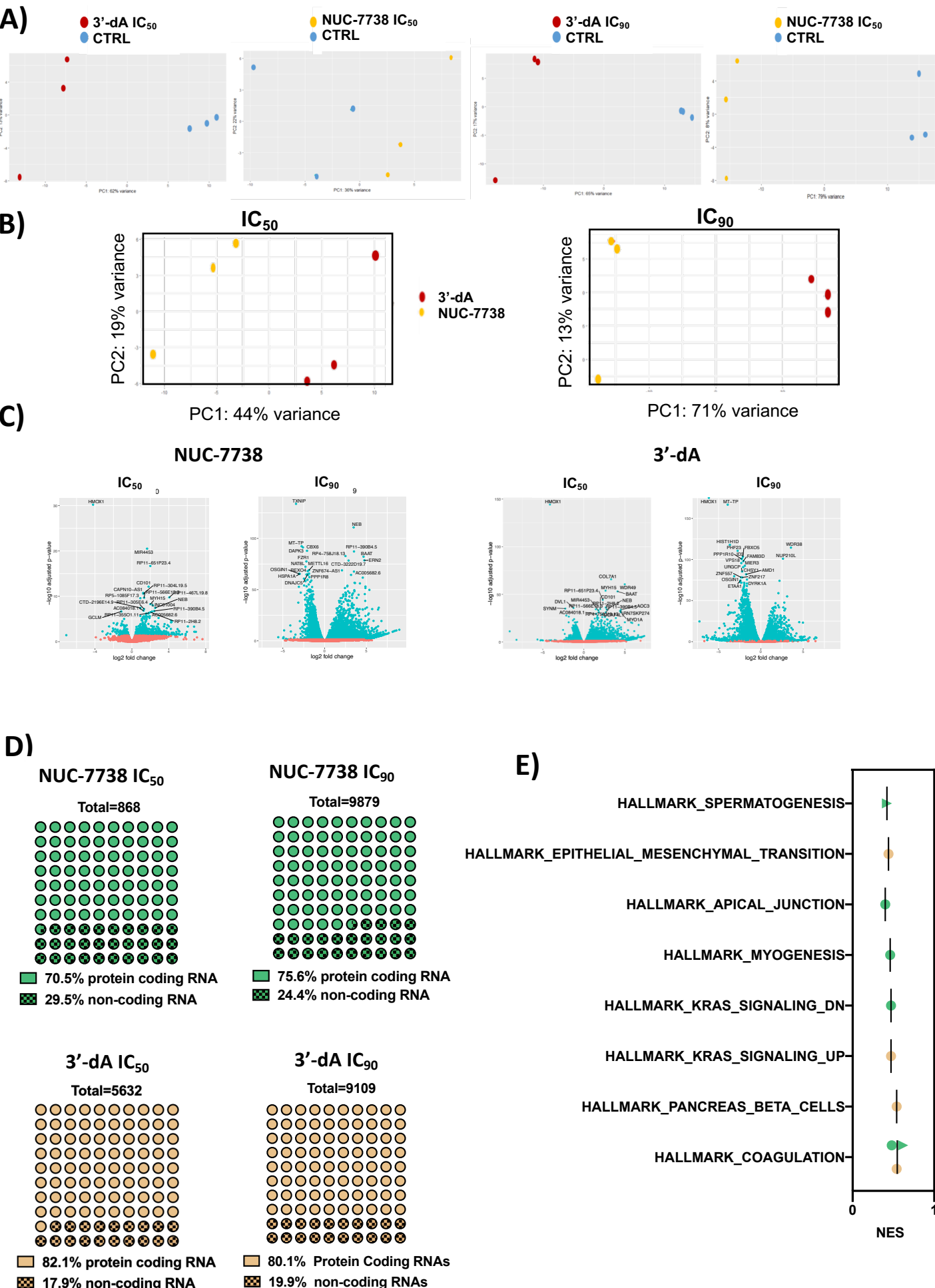

Supplement: Supplementary Figure 4 — Figure S4 related to figure 4: Transcriptional profiling of 3'-dA and NUC-7738 treated HAP1 cells. Principal component analysis from RNA sequencing to compare gene expression variance between NUC-7738 and 3'-dA treated cells versus control (A) and between the two drugs (B). C) Volcano plot analysis obtained from DEseq2 of NUC-7738 and 3'-dA treated cells at two different concentrations. Significant hits (padj<0.05) are highlighted in purple. Non-significant hits are in red. Annotation of TOP20 hits according to adjusted p value are given. D) Relative proportion of protein coding and non-coding RNAs within the detected transcripts. E) Summary of enriched upregulated REACTOME pathways obtained from NUC-7738 and 3'-dA treated cells. Green and orange indicate NUC-7738 and 3'-dA, respectively. Circle and triangle represent dosing at IC50 and IC90, respectively. [file 10780432ccr211652-sup-265190_3_supp_7351756_qy7kv7.pdf]

Figure S5

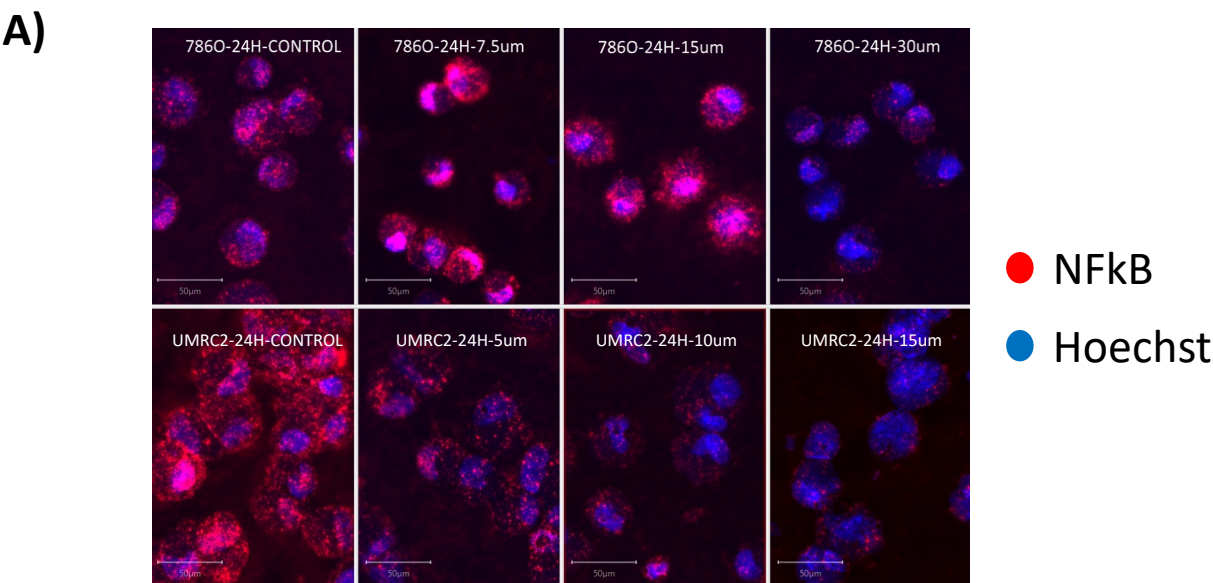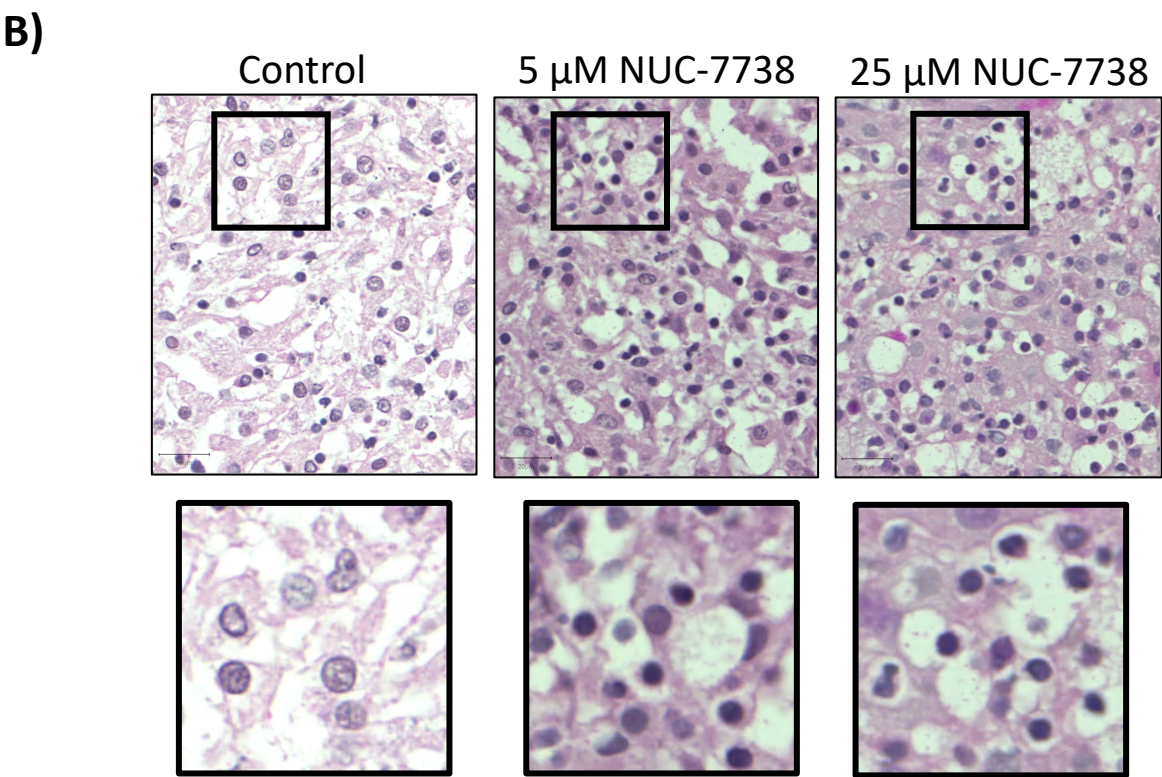

Supplement: Supplementary Figure 5 — Figure S5 related to figure 5: NUC-7738 down-regulates cell survival pathways and induces apoptosis. A) A) Renal 786-O and UM-RC-2 cells were treated with NUC-7738 for 24 hours and stained for NF-kB p65 and nuclear dsDNA (DAPI). P65 was seen in the nucleus of controls but disappeared after treatment with NUC-7738. B) Haematoxylin and Eosin (H&E) staining of ex vivo tissue treated with NUC-7738 for 24 hours. H&E examination showed increased numbers of shrunken pyknotic nuclei corresponding to caspase 3 positive cells. [file 10780432ccr211652-sup-265190_3_supp_7351757_qyskvs.pdf]

Figure S6

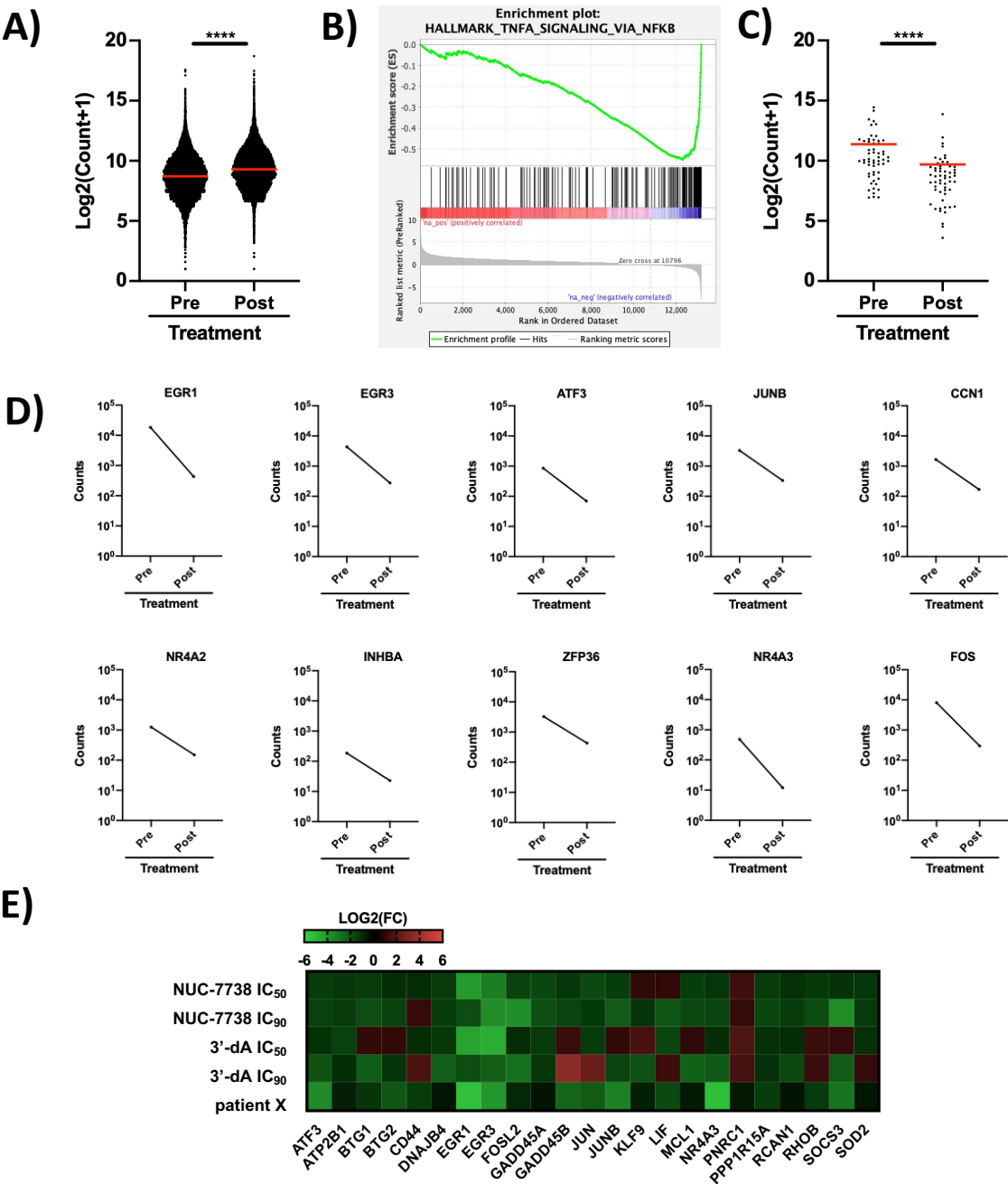

Supplement: Supplementary Figure 6 — Figure S6 related to figure 6: Transcriptomic profiling of post-treatment biopsy taken from one patient with melanoma. A) Violine plots of expression of genes pre- and post-treatment for all genes which showed, in at least one condition, more than 100 counts. B) Enrichment plot for TNF alpha signalling via NF-kB pathway (NES = -3.73, FDR=0). C) Expression of core gene set contributing to the leading edge of NF-kB pathway enrichment plot. D) Expressional changes pre-and post-treatment of TOP 10 genes extracted from NF-kB enrichment plot. E) Overlapping genes between all RNAseq data sets obtained during this study, stratified for NF-kB leading edge genes only. [file 10780432ccr211652-sup-265190_3_supp_7351758_qy4kv4.pdf]
