# Supplementary material for: The Novel Nucleoside Analogue ProTide NUC-7738 Overcomes Cancer Resistance Mechanisms In Vitro and in a First-In-Human Phase I Clinical Trial
Source: Clin Cancer Res. 2021 Dec 1;27(23):6500–13. doi: 10.1158/1078-0432.CCR-21-1652 (PMC9401491; doi:10.1158/1078-0432.CCR-21-1652)
Supplement: Table S1 — related to Figure 1: NUC-7738 has cytotoxic activity [file 10780432ccr211652-sup-265190_3_supp_7356179_qyhshs.docx]

|  | AGS | A498 | ACHN | CAKI-1 | NCI-786 | UO-31 | 501MEL | Tera-1 | SK-OV3 |
| --- | --- | --- | --- | --- | --- | --- | --- | --- | --- |
| NUC-7738 | 11.16 (±1.1) | 48.54 (±19.39) | 54.7 (±16.49) | 32.97 (±23.3) | 49.53 (±6.45) | 42.16 (±2.59) | 10.86 (±1.35) | 4.36 (± 0.86) | 16.57 (n/a) |
| 3’-dA | 460 (±93.3) | 478.8 (±86.5) | 393 (160.6) | 291 (±3.2) | 180.7 (±22.43) | 384.7 (±144.0) | 137 (n/a) | 200.02 (±13.7) | 360 (n/a) |

**Table S1 related to Figure 1: NUC-7738 has cytotoxic activity.** Determination of IC_50_ of 3’-dA or NUC-7738 in gastric, renal, melanoma and ovarian cancer cell lines. Mean and SEM of at least three biological replicates is shown (where no SEM is shown, only 2 biological replicates were performed) in this summary table of Figure 2C. N/A means that no SEM was calculated.
