# Supplementary material for: The Novel Nucleoside Analogue ProTide NUC-7738 Overcomes Cancer Resistance Mechanisms In Vitro and in a First-In-Human Phase I Clinical Trial
Source: Clin Cancer Res. 2021 Dec 1;27(23):6500–13. doi: 10.1158/1078-0432.CCR-21-1652 (PMC9401491; doi:10.1158/1078-0432.CCR-21-1652)
Supplement: Table S2 — related to Figure 2: Genome wide haploid genetic screen identifies genes necessary for the activity of 3'-dA and NUC-7738 [file 10780432ccr211652-sup-265190_3_supp_7351774_qyzl2z.docx]

| **3’-dA (µM)** | | **NUC-7738 (µM)** | | | | | |
| --- | --- | --- | --- | --- | --- | --- | --- |
| **375** | | **50** | | **100** | | **125** | |
| ***gene name*** | ***padj*** | ***gene name*** | ***padj*** | ***gene name*** | ***padj*** | ***gene name*** | ***padj*** |
| ADK | 2.94E-304 | EIF2AK4 | 1.01E-17 | CREBBP | 1.32E-45 | HINT1 | 4.30E-74 |
| NUDT2 | 2.65E-153 | CREBBP | 1.48E-15 | SPEN | 1.25E-34 | CERCAM | 2.18E-53 |
| PITPNC1 | 1.03E-03 | APAF1 | 2.53E-15 | SGF29 | 9.20E-34 | GLG1 | 8.75E-41 |
| UBE2G1 | 9.16E-06 | SGF29 | 4.94E-14 | STX18 | 6.64E-33 | MEGF9 | 1.51E-13 |
| KIF3B | 8.83E-05 | NF2 | 1.05E-12 | HINT1 | 4.07E-32 | SMARCA4 | 3.67E-13 |
| CUX1 | 6.97E-03 | STX18 | 3.16E-12 | SMARCA4 | 1.57E-27 | SMARCC1 | 2.66E-10 |
| PPEF1 | 1.34E-10 | EBF3 | 2.20E-11 | EIF2AK4 | 9.45E-19 | PREP | 4.52E-08 |
| CCDC109B | 1.06E-09 | SPEN | 3.55E-11 | SETD1B | 3.25E-17 | SGF29 | 4.47E-04 |
| CDH1 | 5.87E-17 | TAOK1 | 2.98E-10 | DLEC1 | 6.37E-17 | GNPTAB | 7.96E-04 |
| KIF9 | 3.16E-02 | SETD1B | 1.44E-09 | USP14 | 1.19E-14 | WDR33 | 1.66E-03 |
| PTPN9 | 2.64E-02 | SMARCA4 | 4.02E-09 | ILF3 | 2.74E-14 | ELP4 | 7.15E-03 |
| HERC4 | 3.18E-05 | DLEC1 | 7.87E-09 | BTLA | 5.40E-14 | KRT73 | 7.22E-03 |
| GRAMD1C | 4.72E-02 | EPC2 | 1.97E-08 | NF2 | 3.40E-12 | SMYD4 | 7.22E-03 |
| TBC1D14 | 3.98E-03 | HINT1 | 5.25E-08 | DDX6 | 4.65E-12 | NLGN4Y | 8.84E-03 |
| WNK3 | 8.77E-06 | ANKRD13C | 5.25E-08 | UBASH3B | 9.82E-12 | CASC5 | 1.24E-02 |
| BTLA | 1.82E-10 | MAP4K3 | 7.24E-08 | GCN1 | 8.37E-11 | BAIAP2L1 | 1.49E-02 |
| TEX11 | 3.11E-06 | UBASH3B | 8.81E-08 | ZNF793 | 2.11E-10 | PHACTR1 | 1.49E-02 |
| DCST1 | 1.52E-02 | ZNF793 | 1.25E-07 | CDH1 | 2.85E-10 | POMZP3 | 1.49E-02 |
| TAX1BP1 | 2.89E-02 | RPS25 | 2.77E-07 | CTCF | 2.85E-10 | GRIP1 | 1.53E-02 |
| SLC29A1 | 7.05E-10 | USP14 | 1.38E-06 | ARID1A | 9.29E-10 | MYO16 | 1.60E-02 |

**Table S2** **related to Figure 2: Genome wide haploid genetic screen identifies genes necessary for the activity of 3’-dA and NUC-7738.** Top 20 genes from each treatment are sorted according to their p-value.
