# Supplementary material for: The Novel Nucleoside Analogue ProTide NUC-7738 Overcomes Cancer Resistance Mechanisms In Vitro and in a First-In-Human Phase I Clinical Trial
Source: Clin Cancer Res. 2021 Dec 1;27(23):6500–13. doi: 10.1158/1078-0432.CCR-21-1652 (PMC9401491; doi:10.1158/1078-0432.CCR-21-1652)
Supplement: Table S3 — related to Figure 2: Genome wide haploid genetic screen identifies genes necessary for the activity of 3'-dA and NUC-7738. [file 10780432ccr211652-sup-265190_3_supp_7351775_qygl2z.docx]

| **gene** **name** | **average** **rank** | **gene** **name** | **average** **rank** | **gene** **name** | **average** **rank** | **gene** **name** | **average** **rank** | **gene** **name** | **average** **rank** |
| --- | --- | --- | --- | --- | --- | --- | --- | --- | --- |
| SMARCA4 | 8.5 | HTR7 | 32 | HOMER2 | 70.5 | PCMTD2 | 116 | ZNF91 | 330.5 |
| NF2 | 9 | ZNF578 | 35.5 | CNTNAP5 | 73 | BCL2L1 | 133.5 | B4GALNT2 | 395 |
| DLEC1 | 10.5 | TRPC7 | 36.5 | LHFPL3 | 79.5 | LIPC | 146.5 | REPS2 | 603.5 |
| UBASH3B | 16 | PBX4 | 49 | HERC4 | 87.5 | CDC123 | 147 | ZNF611 | 645.5 |
| BTLA | 16.5 | SLC1A2 | 55.5 | MAP1LC3A | 96.5 | KIF3B | 154.5 | PRTN3 | 1045.5 |
| ZNF793 | 17.5 | ADGRD1 | 59 | ST8SIA1 | 104 | RAD54L2 | 226.5 | CDH18 | 1227 |
| ANKRD13C | 20 | TEX11 | 61.5 | HMCN1 | 105.5 | DNTT | 228 | PITPNC1 | 1454 |
| CDH1 | 24 | CCDC109B | 65.5 | PARVG | 109 | FAM46D | 235.5 | UBE2G1 | 1631 |
| FLVCR2 | 29 | TSPAN16 | 68 | NOX5 | 111 | WDPCP | 255.5 | CACNA1A | 2271 |
| PPEF1 | 29.5 | PLEC | 68 | EPHB1 | 113 | WNK3 | 273 |  |  |

**Table S3** **related to Figure 2: Genome wide haploid genetic screen identifies genes necessary for the activity of 3’-dA and NUC-7738.** Top 20 genes found in all four treatments. Gene hits sorted according their average rank out of the 3 different NUC-7738 concentrations.
