# Supplementary material for: The Novel Nucleoside Analogue ProTide NUC-7738 Overcomes Cancer Resistance Mechanisms In Vitro and in a First-In-Human Phase I Clinical Trial
Source: Clin Cancer Res. 2021 Dec 1;27(23):6500–13. doi: 10.1158/1078-0432.CCR-21-1652 (PMC9401491; doi:10.1158/1078-0432.CCR-21-1652)
Supplement: Supplementary Materials — NMR spectra for synthesised compounds [file 10780432ccr211652-sup-265190_3_supp_7366177_qyr1d3.docx]

**Supplementary Materials**

**NMR spectra for synthesised compounds:**

**10** as a sticky solid:

^1^H NMR (500 MHz, CDCl_3_) δ_H_ 8.36 (s, 1H, H8), 8.34 (s, 1H, H2), 6.03 (d, *J* = 1.2 Hz, 1H, H1’), 5.54 (br s, 2H, NH_2_), 4.66-4.63 (m, 1H, H2’), 4.61-4.55 (m, 1H, H4’), 4.14 (dd, *J* = 11.7, 2.7 Hz, 1H, H5’), 3.80 (dd, *J* = 11.7, 2.7 Hz, 1H, H5’), 2.32-2.25 (m, 1H, H3’), 1.91-1.85 (m, 1H, H3’), 0.97 (s, 9H, *t*Bu), 0.92 (s, 9H, *t*Bu), 0.17 (s, 3H, CH_3_), 0.16 (s, 3H, CH_3_), 0.15 (s, 3H, CH_3_), 0.10 (s, 3H, CH_3_).

**11** as a white foam:

^1^H NMR (500 MHz, CDCl_3_) δ_H_ 8.45 (s, 1H, H8), 7.97 (s, 1H, H2), 6.29 (br s, 2H, NH_2_), 5.74 (d, *J* = 6.0 Hz, 1H, H1’), 5.19-5.12 (m, 1H, H2’), 4.65-4.60 (m, 1H, H4’), 4.11 (dd, *J* = 13.0, 1.5 Hz, 1H, H5’), 3.67 (dd, *J* = 12.5, 1.5 Hz, 1H, H5’), 2.72-2.62 (m, 1H, H3’), 2.37-2.26 (m, 1H, H3’), 1.48 (d, *J* = 7.0 Hz, 1H, OH-5’), 0.91 (s, 9H, *t*Bu), 0.00 (s, 3H, CH_3_), -0.12 (s, 3H, CH_3_).

**12** as a white solid:

^31^P NMR (202 MHz, CD_3_OD) δ_P_ 2.89, 2.98.^1^H NMR (500 MHz, CDCl_3_) δ_H_ 8.44 (s, 0.5H, H8), 8.43 (s, 0.5H, H8), 8.20 (s, 0.5H, H2), 8.19 (s, 0.5H, H2), 7.49-7.20 (m, 10H, Ar), 6.23 (br s, 2H, NH2), 6.05 (d, *J* = 9.0 Hz, 0.5H, H1’), 6.03 (d, *J* = 9.0 Hz, 0.5H, H1’), 4.98-4.89 (m, 1H, H2’), 4.81-4.72 (m, 1H, H3’), 4.56-4.32 (m, 2H, H5’), 4.26-4.16 (m, 1H, C*H*CH_3_), 2.32-1.99 (m, 2H, H3’), 1.01 (s, 4.5H, *t*Bu), 1.00 (s, 4.5H, *t*Bu), 0.23 (d, *^3^J*_C-P_ = 7.0 Hz, 1.5H, CHC*H_3_*), 0.21 (d, *^3^J*_C-P_ = 7.0 Hz 1.5H, CHC*H_3_*), 0.20 (s, 1.5H, CH_3_), 0.19 (s, 1.5H, CH_3_).

**7** as a white solid:

^31^P NMR (202 MHz, CD_3_OD) δ_P_ 3.91, 3.73.^1^H NMR (500 MHz, CDCl_3_) δ_H_ 8.26 (s, 0.5H, H8), 8.24 (s, 0.5H, H8), 8.22 (s, 0.5H, H2), 8.21 (s, 0.5H, H2), 7.34-7.25 (m, 7H, Ar), 7.21-7.13 (m, 3H, Ar), 6.01 (d, *J* = 1.5 Hz, 0.5H, H1’), 6.00 (d, *J* = 1.5 Hz, 0.5H, H1’), 5.15-5.04 (m, 2H, C*H_2_*Ph), 4.73-4.63 (m, 2H, H2’, H4’), 4.43-4.35 (m, 1H, H5’), 4.27-4.20 (m, 1H, H5’), 4.03-3.91 (m, 1H, C*H*CH_3_), 2.35-2.28 (m, 1H, H3’), 2.09-2.02 (m, 1H, H3’), 1.32 (d, *J* = 7.4 Hz, 1.5 H, CHC*H_3_*), 1.28 (d, *J* = 7.4 Hz, 1.5 H, CHC*H_3_*). ^13^C NMR (125 MHz, CD_3_OD) δ_C_ 174.84 (d, *^3^J*_C-P_ = 4.5 Hz, C=O), 174.63 (d, *^3^J*_C-P_ = 4.5 Hz, C=O), 157.32 (C-6), 157.31 (C-6), 153.86 (C-2), 153.84 (C-2), 152.13 (C-4), 152.07 (C-4), 150.20 (C-Ar), 150.18 (C-Ar), 140.47 (C-8), 137.26 (C-Ar), 137.19 (C-Ar), 130.76 (CH-Ar), 130.74 (CH-Ar), 129.57 (CH-Ar), 129.32 (CH-Ar), 129.31 (CH-Ar), 129.29 (CH-Ar), 129.26 (CH-Ar), 126.16 (CH-Ar), 126.14 (CH-Ar), 121.46 (d, *^3^J*_C-P_ = 4.7 Hz, CH-Ar), 121.38 (d, *^3^J*_C-P_ = 4.7 Hz, CH-Ar) 120.54 (C-5), 120.53 (C-5), 93.24 (C-1’), 93.18 (C-1’), 80.43 (d, *^3^J*_C-P_ = 3.6 Hz, C-4’), 80.36 (d, *^3^J*_C-P_ = 3.6 Hz, C-4’), 76.62 (C-2’), 68.62 (d, *^2^J*_C-P_ = 5.3 Hz, C-5’), 68.30 (d, *^2^J*_C-P_ = 5.3 Hz, C-5’), 67.95 (*C*H_2_Ph), 67.92 (*C*H_2_Ph), 51.74 (*C*HCH_3_), 51.60 (*C*HCH_3_), 34.91 (C-3’), 34.70 (C-3’), 20.45 (d, *^3^J*_C-P_ = 7.0 Hz, CH*C*H_3_), 20.28 (d, *^3^J*_C-P_ = 7.0 Hz, CH*C*H_3_).
